# Isoniazid Linked to Sulfonate Esters via Hydrazone Functionality: Design, Synthesis, and Evaluation of Antitubercular Activity

**DOI:** 10.3390/ph15101301

**Published:** 2022-10-21

**Authors:** Ebru Koçak Aslan, Muhammed İhsan Han, Vagolu Siva Krishna, Rasoul Tamhaev, Cagatay Dengiz, Şengül Dilem Doğan, Christian Lherbet, Lionel Mourey, Tone Tønjum, Miyase Gözde Gündüz

**Affiliations:** 1 Department of Pharmaceutical Chemistry, Faculty of Pharmacy, Hacettepe University, Sıhhiye, Ankara 06100, Turkey; 2 Department of Pharmaceutical Chemistry, Faculty of Pharmacy, Erciyes University, Kayseri 38039, Turkey; 3 Unit for Genome Dynamics, Department of Microbiology, University of Oslo, 0316 Oslo, Norway; 4 LSPCMIB, UMR-CNRS 5068, Université Paul Sabatier-Toulouse III, 118 Route de Narbonne, CEDEX 9, 31062 Toulouse, France; 5 Institut de Pharmacologie et de Biologie Structurale, Université Toulouse III—Paul Sabatier, Centre National de la Recherche Scientifique, 31077 Toulouse, France; 6 Department of Chemistry, Middle East Technical University, Ankara 06800, Turkey; 7 Department of Basic Sciences, Faculty of Pharmacy, Erciyes University, Kayseri 38039, Turkey; 8 Unit for Genome Dynamics, Department of Microbiology, Oslo University Hospital, 0424 Oslo, Norway

**Keywords:** antimycobacterial, *Mycobacterium tuberculosis*, drug-resistant clinical isolates, molecular modeling, InhA

## Abstract

Isoniazid (INH) is one of the key molecules employed in the treatment of tuberculosis (TB), the most deadly infectious disease worldwide. However, the efficacy of this cornerstone drug has seriously decreased due to emerging INH-resistant strains of *Mycobacterium tuberculosis* (*Mtb*). In the present study, we aimed to chemically tailor INH to overcome this resistance. We obtained thirteen novel compounds by linking INH to in-house synthesized sulfonate esters via a hydrazone bridge (**SIH1–SIH13**). Following structural characterization by FTIR, ^1^H NMR, ^13^C NMR, and HRMS, all compounds were screened for their antitubercular activity against *Mtb* H37Rv strain and INH-resistant clinical isolates carrying *katG* and *inhA* mutations. Additionally, the cytotoxic effects of **SIH1–SIH13** were assessed on three different healthy host cell lines; HEK293, IMR-90, and BEAS-2B. Based on the obtained data, the synthesized compounds appeared as attractive antimycobacterial drug candidates with low cytotoxicity. Moreover, the stability of the hydrazone moiety in the chemical structure of the final compounds was confirmed by using UV/Vis spectroscopy in both aqueous medium and DMSO. Subsequently, the compounds were tested for their inhibitory activities against enoyl acyl carrier protein reductase (InhA), the primary target enzyme of INH. Although most of the synthesized compounds are hosted by the InhA binding pocket, **SIH1–SIH13** do not primarily show their antitubercular activities by direct InhA inhibition. Finally, in silico determination of important physicochemical parameters of the molecules showed that **SIH1–SIH13** adhered to Lipinski’s rule of five. Overall, our study revealed a new strategy for modifying INH to cope with the emerging drug-resistant strains of *Mtb*.

## 1. Introduction

*Mycobacterium tuberculosis* (*Mtb*) is the primary causative agent of the contagious disease tuberculosis (TB) that has devastated humankind for centuries [1]. TB is one of the leading infectious agent-related causes of mortality, as confirmed by the data presented in the latest World Health Organization (WHO) Global TB Report [2]. The successful control of TB by the current chemotherapeutic regimen, mainly consisting of four first-line drugs (isoniazid, rifampicin, pyrazinamide, and ethambutol) [3], is often endangered by emerging drug-resistant *Mtb* strains [4].

**Figure 1:**
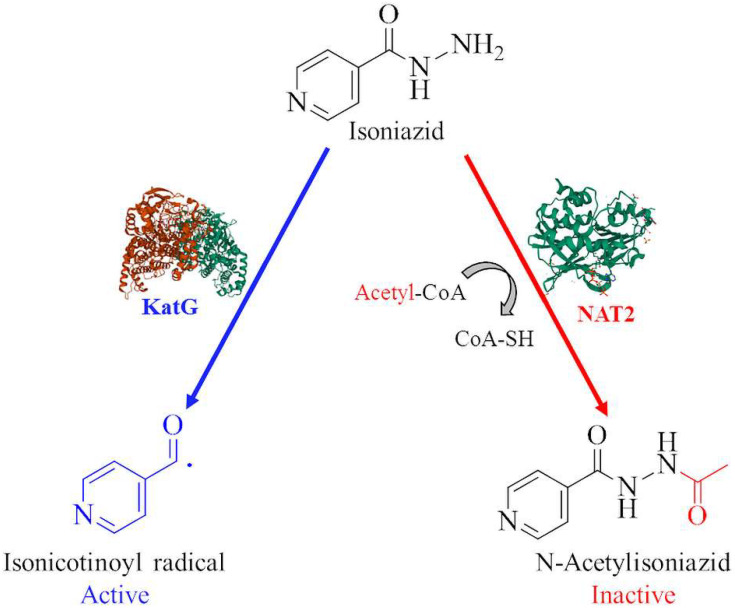
Schematic depiction of pathways leading to activation and inactivation of INH.

Isoniazid (isonicotinic acid hydrazide, INH) is the cornerstone drug of modern TB therapy [5]. As a prodrug, INH needs to be oxidized by the enzyme catalase-peroxidase (KatG) to inhibit its biological target enoyl-acyl carrier protein reductase (InhA), a critical enzyme employed in the biosynthesis of mycolic acids [6,7]. Therefore, the clinical efficacy of INH in the treatment of drug-resistant TB is restricted due to mutations in *katG* or *inhA* and its promoter [8,9]. Furthermore, in humans, INH is metabolized and in this way deactivated by *N*-acetyltransferase type 2 (NAT2). This enzyme is responsible for the acetylation of the terminal nitrogen of the hydrazide group of INH using acetyl coenzyme A (acetyl-CoA) as the acetyl donor [10]. Due to genetic polymorphism in NAT2, variable acetylation rates arise, resulting in variations in blood drug concentrations among patients treated with INH [11]. These metabolic steps, which ultimately result in the activation and deactivation of INH, are schematically presented in Figure 1.

**Figure 2:**
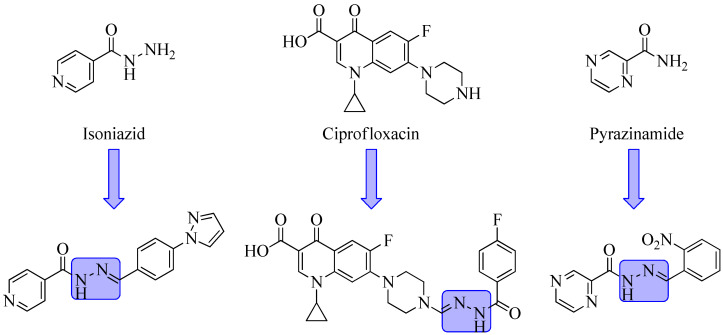
Biologically active hydrazone derivatives of marketed anti-TB drugs.

Due to their commonly reported anti-TB properties, hydrazone-possessing molecules are of considerable interest [12]. Particularly, the synthesis of hydrazone derivatives of the current anti-TB drugs such as isoniazid [13], ciprofloxacin [14], and pyrazinamide [15] is a frequently applied antimycobacterial drug design strategy (Figure 2). Therefore, hydrazone is considered to be an excellent linker fragment for newly-designed antitubercular agents.

As one of the most common ways to design and develop new drugs, molecular hybridization involves merging two or more pharmacophores in a single chemical entity, either directly or by using spacer units. The main goal of this strategy is to improve the biological profile while reducing side effects as much as possible [16,17].

As previously indicated, INH is a significant component of the standardized regimens for TB treatment recommended by WHO. However, inappropriate treatment or poor patient compliance with the therapy often leads to the emergence of INH-resistant strains of *Mtb* [18]. Thus, tailoring the chemical structure of INH is accepted as a logical approach for designing new antitubercular compounds to overcome emerging resistance. Notably, modifications that allow INH to directly show its antimycobacterial effect without necessitating KatG activation or avoid the hydrazide functionality from acetylation can provide a new avenue for antitubercular drug development [19].

**Figure 3:**
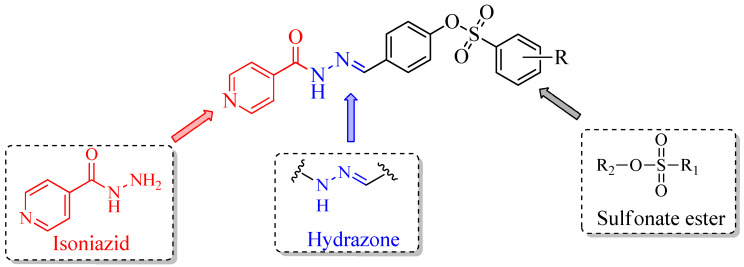
Design strategy of the studied molecules **SIH1–SIH13**.

Prompted by these considerations, we set out with the goal of developing new antitubercular agents (**SIH1–SIH13**) by linking INH to various sulfonate esters that were also reported to exhibit antitubercular activities [20,21] via hydrazone bridge (Figure 3). When choosing the substituents on the sulfonate moiety, we mainly modified the *para* position of the terminal phenyl ring with various different groups including alkyl, aryl, acyl, halogen, and nitro. In this way, we made sure that the antitubercular activity was due to the substituent type independent from the position. Additionally, we carried out some additional chemical modifications (introducing two halogens, adding more alkyl groups, condensing phenyl to a heterocyclic ring, or changing the position of nitro group) to see how they will affect antimycobacterial activity and to establish preliminary structure-activity relationships for this class of compounds. Subsequently, we tested the antimycobacterial potency of these compounds against the reference *Mtb* strain H37Rv as well as against INH-resistant clinical isolates carrying *katG* and *inhA* promoter mutations.

## 2. Results and Discussion

### 2.1. Chemistry

**Scheme 1:**
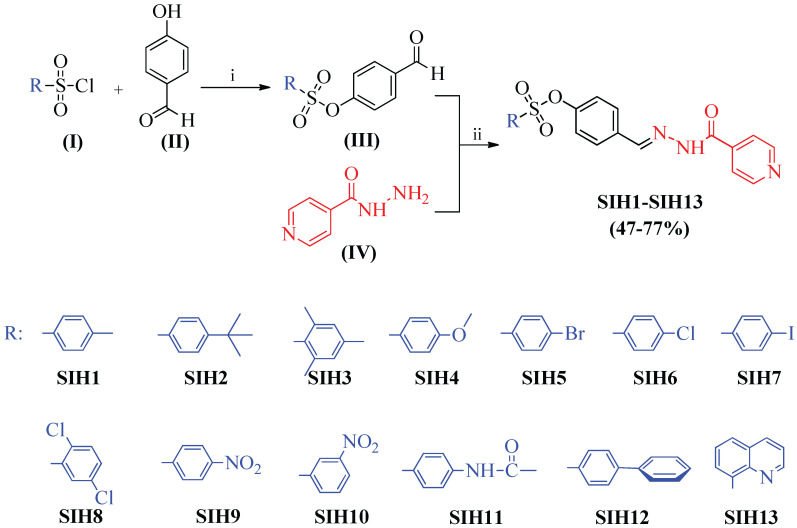
Reagents and conditions: (**i**) TEA/DMF, rt, (**ii**) EtOH/AcOH, reflux.

The synthesis and chemical structures of the new isoniazid derivatives (**SIH1–SIH13)** are outlined in Scheme 1. The key intermediates, 4-formylphenyl benzenesulfonates, (**III**) were prepared through the reaction of aromatic sulfonyl chlorides (**I**) with 4-hydroxybenzaldehyde (**II**) in DMF in the presence of triethyl amine (TEA). Our target compounds (**SIH1–SIH13**) were obtained in 47–77 % yield by refluxing 4-formylphenyl benzenesulfonates (**III**) and isoniazid (**IV**) in ethanol in the presence of a catalytic amount of glacial acetic acid.

The structures of the newly synthesized compounds (**SIH1–SIH13)** were confirmed by their infrared (IR), ^1^H NMR, ^13^C NMR, and HRMS spectral data. In IR spectra, **SIH1–SIH13** were characterized by the appearance of absorption bands in the range of 3396–3248 cm^−1^, 1607–1596 cm^−1^, and 1676–1651 cm^−1^ due to N-H, C=N, and C=O stretching vibrations, respectively. Besides these, two characteristic bands for the SO_2_ stretches were observed in the ranges of 1352–1335 and 1143–1152 cm^−1^. Furthermore, ^1^H NMR spectra of the compounds displayed two singlets at 12.21–12.11 ppm (O=C-N**H**) and 8.46–8.37 ppm (N=C**H**), respectively. According to the literature, the presence of a singlet downfield resonating (8.46–8.37 ppm) N=C**H** signal, exclusively accounts for the formation of *E*-isomers [22]. In addition, ^13^C NMR spectra of the molecules showed a smaller number of resonances than the exact number of carbons due to the overlapped peaks in the aromatic field. The ^13^C NMR spectra exhibit the **C**=O signal at 162.4–162.1 ppm and **C**=N signal at 140.7–140.8 ppm, confirming the *N*-acylhydrazone skeleton in the chemical structures of **SIH1–SIH13**. ^1^H NMR, ^13^C NMR, HRMS, and FTIR spectra of the final compounds are provided in Supplementary Material.

### 2.2. Antitubercular Activity Evaluation and Cytotoxicity Assessment

We tested the antimycobacterial activity of all the compounds obtained against *Mtb* H37Rv and two INH-resistant clinical isolates (*katG* and *inhA* promoter mutants) by applying the Microplate Alamar Blue Assay (MABA) protocol. As toxicity to healthy host cells is a significant barrier to launching new antitubercular drugs, we additionally assessed the cytotoxicity of our compounds against three different cell lines: human embryonic kidney (HEK) 293, human lung cells IMR-90, and human epithelial bronchus cells BEAS-2B. Minimum inhibitory concentration (MIC) values against three *Mtb* strains tested as well as toxicity data of **SIH1–SIH13** are provided in Table 1.

Based on the activity analyses, four compounds (**SIH1**, **SIH4**, **SIH12**, and **SIH13**) exhibited MIC values of 0.31 μM against *Mtb* H37Rv. The other molecules in this series were also efficient as antitubercular agents with MIC values of 0.62 μM. As expected, the MIC values of INH against two defined INH-resistant (*inhA* promoter mutant and *katG* S315T mutant) clinical isolates were found to be higher than the value obtained for INH-sensitive *Mtb* H37Rv. Against the *inhA* mutant INH-resistant *Mtb* clinical isolate, **SIH1**, **SIH2**, and **SIH4** were effective with MIC values of 1.56. Except for two compounds, **SIH5** and **SIH6** (MIC = 6.25 μM), the remaining compounds displayed antimycobacterial activity with MIC values of 3.12 μM. **SIH1–SIH13** were also screened against the other INH-resistant *Mtb* isolate with *katG* mutation. Among them, **SIH4** was the most effective compound with a MIC value of 6.25 μM which was the same value as calculated for INH. Excluding **SIH6** and **SIH7**, other molecules also represented antitubercular activity with MIC values ranging between 12.5 and 50 μM against *katG* mutant *Mtb* clinical isolate.

When the molecules were examined in terms of their chemical structures, it is noteworthy that **SIH4** with *p*-methoxy group on its phenyl ring was the most attractive compound showing the lowest MIC values against all the *Mtb* strains tested. Moreover, the other most active compounds against *Mtb* H37Rv carry a methyl (**SIH1**) or a phenyl (**SIH12**) group, or a condensed pyridine ring (**SIH13**) on their terminal phenyl rings. For the inhibition of *inhA* mutant *Mtb* strains, methyl (**SIH1**), *t*-butyl (**SIH2**), and methoxy (**SIH4**) on the *para* position of the phenyl ring were the most preferential substituents. As extensive molecular modifications were carried out on the phenyl ring of sulfonate esters, it can be concluded that the changes in substituent types, positions, and volumes can easily be tolerated on this scaffold for antitubercular activity.

Furthermore, based on the cytotoxicity data obtained from the colorimetric MTT assay, the compounds were found to be non-toxic against lung cell lines IMR-90 and BEAS-2B. Only **SIH12** was slightly toxic towards IMR-90 with IC_50_ value of 57.7 μM. Against HEK293, except for **SIH1**, **SIH4**, **SIH12**, and **SIH13** that were moderately toxic, our compounds did not present cytotoxicity. As TB predominantly is a respiratory disease, it is critical that we identified effective antimycobacterial drug candidates with favorable toxicity profiles, especially against the lung cells included in this study.

### 2.3. Stability Studies in Water and DMSO

Imine formations are well-known reversible condensation reactions [23]. Solvent type is one of the factors that need to be taken into account while assessing the stability of imines [24]. In the presence of water, the equilibrium may shift in favor of precursors and the target compounds to be tested in activity assays may be misinterpreted. Therefore, we tested the stability of our compounds under aqueous conditions to confirm the stability of the hydrazone functionality. In this way, we could make sure that the antitubercular activity of the molecules studied is not due to INH as one of the hydrolysis products of our compounds. Furthermore, the stability of the selected compounds was also assessed in DMSO, the solvent used in the biological experiments.

**Figure 4:**
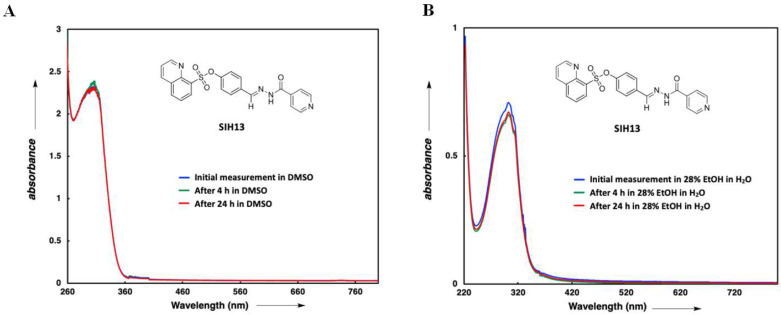
Stability assessment of **SIH13** in DMSO (**A**) and ethanol-water mixture (**B**).

Accordingly, we performed stabilization tests for four selected compounds (**SIH1**, **SIH4**, **SIH12**, and **SIH13**) in both aqueous medium and DMSO by using UV/Vis spectroscopy. These representative compounds were selected based on their low MIC values against *Mtb* H37Rv strain. Samples with a concentration of 6.0 × 10^–5^ M were prepared in DMSO and a 28% ethanol-water mixture. The change in λ_max_ values throughout 24 h in the selected solvents was monitored to assess the stability of the hydrazone functionality. As expected, no significant change in λ_max_ values was observed for the compounds. UV/Vis absorption spectra of **SIH13** in DMSO and ethanol-water are provided in Figure 4. The results for **SIH1**, **SIH4**, and **SIH12** are reported in the Supplementary Material.

### 2.4. InhA Inhibition

INH principally targets the InhA enzyme that is responsible for the biosynthesis of long-chain fatty acids in *Mtb*. Fatty acids, particularly mycolic acids, play a crucial role in the survival of *Mtb* since they are structural components of the bacterial cell wall [25]. Following the activation by KatG, INH produces a reactive isonicotinoyl-radical intermediate that quickly forms a covalent adduct with the InhA cofactor NADH [6]. As mutations in *katG* reduce the activation of INH and thus yield the most prevalent type of INH resistance, developing direct InhA inhibitors that do not require KatG activation appears as an effective and rational strategy to overcome this resistance problem [26]. Therefore, we screened our INH-based compounds against InhA to determine if they show their antimycobacterial activity via direct inhibition of this enzyme (Table 2).

Although most of the molecules are recognized by the binding pocket of InhA, our compounds do not primarily represent their antitubercular activity by inhibiting InhA enzyme directly. **SIH7** was found to be a moderate inhibitor of InhA with 41% inhibition. As the main structural difference between the tested compounds is the substituent type and position on the phenyl ring of the sulfonate moiety, it is noteworthy that introducing iodine as a bulky and lipophilic halogen can be an important modification to bind to the active site of the enzyme. These data can be used useful for the design of effective antimycobacterial INH derivatives that are also capable of inhibiting InhA.

### 2.5. Molecular Modeling Studies

#### 2.5.1. Molecular Docking

The active pocket of InhA is large enough to accommodate various direct inhibitors with different chemical scaffolds such as diphenyl ethers, 4-hydroxy-2-pyridones, benzamides, and thiadiazoles [27,28]. Despite this flexibility, in almost every case, the direct inhibitors block the enzyme via forming two key hydrogen bonds to Tyr158 and nicotinamide adenine dinucleotide (NAD) cofactor of InhA. Additional hydrophobic interactions are also required with the terminal lipophilic pockets of the binding site [29]. To rationalize the obtained data and explain the binding mode of our compounds to InhA, we carried out in silico studies. We docked **SIH7** into the binding pocket of InhA and compared its interactions to those of GEQ/Genz-10850, a direct inhibitor co-crystallized within the enzyme. The binding modes and the interactions of the compounds within the active pocket of InhA are provided in Supplementary Materials.

#### 2.5.2. In Silico Prediction of Drug-Likeness

The unsuitable physicochemical profiles of drug candidate molecules can be a serious obstacle in the drug development process for the conversion of bioactive compounds into drugs. Thus, we performed theoretical calculations to identify drug-like properties of our compounds. We started with computing the descriptors that constitute the Lipinski’s rule of five. This rule finds out if a chemical compound with preferable biological profile possesses suitable physicochemical properties that make it orally active in humans. Accordingly, to show oral bioavailability, a molecule should not breach more than one of the following requirements: molecular weight (MW) ≤ 500, *n*-octanol/water partition coefficient (log P) ≤ 5, number of hydrogen bond donors (HBD) ≤ 5 and number of hydrogen bond acceptors (HBA) ≤ 10 [30]. Furthermore, we predicted the compounds’ topological polar surface areas (TPSAs) and numbers of rotatable bonds (NRBs), both of which are regarded to be crucial characteristics for predicting the oral bioavailability of novel molecules [31]. The predicted physicochemical descriptors of **SIH1–SIH13** are presented in Table 3.

Based on the estimated parameters, all compounds, except for **SIH7**, were shown to entirely follow Lipinski’s rule of five. **SIH7** displays only one violation with a molecular weight of slightly more than 500. Furthermore, according to the calculated LogP values, the compounds are highly lipophilic compared to INH (LogP = −0.35, calculated in SwissADME). Besides mutations in either *katG* or the *inhA* promoter, this low hydrophobicity of INH is linked to the long duration of TB treatment [32]. The mycolic acid outer layer of *Mtb* is so lipophilic that INH cannot efficiently penetrate and cross it. The permeability of INH in the *Mtb* granuloma is also low due to its hydrophilic character [33]. Therefore, in this study, we succeeded in increasing the lipophilicity of INH with suitable chemical modifications to enhance its ability to diffuse through the cell membrane. The number of rotatable bonds, a significant descriptor of molecular flexibility, should be less than ten to keep conformational changes at minimum during the interactions with the biological targets. **SIH1–SIH13** follow this criterion as they all have ≤10 rotatable bonds. Additionally, TPSA of the compounds varied between 106.10 and 151.92 Å^2^. Most of the clinically used drug molecules have TPSA less than 140–150 Å^2^ [34], so our molecules stay in the acceptable range although the values calculated for **SIH9** and **SIH10** are slightly higher than 150 Å^2^.

Consequently, the physicochemical properties of **SIH1–SIH13** support their potential position as relevant candidates in the antimycobacterial drug development process.

## 3. Material and Methods

### 3.1. Chemistry

#### 3.1.1. Experimental

All the chemicals used in this study were purchased from commercial sources and were used without further purification. The completion of reactions and purities of the final compounds were monitored by thin layer chromatography (TLC) using Silica Gel 60 F254 TLC plate (Merck, Darmstadt, Germany). The R_f_ values of all the synthesized compounds were determined using ethyl acetate:*n*-hexane:methanol (7:2:1) as mobile phase. The melting points were determined by using Thomas Hoover Capillary Melting Point Apparatus (Philadelphia, PA, USA) and were uncorrected. Infrared (IR) spectra were obtained on Perkin Elmer Spectrum BX FT-IR (Beaconsfield, UK) and reported in the range of 4000–400 cm^−1^ using ATR accessory (Pike Technologies) with a diamond ATR crystal. The NMR spectra were recorded by Bruker spectrometer. ^1^H NMR spectra were run at 400 MHz, while ^13^C NMR spectra were run at 100 MHz in deuterated dimethyl sulfoxide (DMSO-*d*_6_). Chemical shifts (*δ*_H_) and (*δ*_C_) are expressed in values (ppm) using the solvent peak as the internal standard. All coupling constant (*J*) values are given in Hertz.

#### 3.1.2. Synthesis

**Preparation of aromatic aldehydes (III):** Sulfonyl chlorides (1 mmol) and 4-hydroxybenzaldehyde (1.1 mmol) were mixed in DMF (20 mL) for 24 h at room temperature in the presence of triethylamine (1.35 mL). After pouring the mixture into ice, the resulting precipitate was filtered, washed with water, dried, and crystallized from ethanol [35].

**General procedure for the synthesis of the target compounds SIH1–SIH13:** A mixture of appropriate 4-formylphenyl substituted benzenesulfonate (0.5 mmol) and isoniazid (0.5 mmol) was refluxed with 5 drops of acetic acid in 15 mL methanol for 8 h. After completion of the reaction, the mixture was cooled and the precipitated solid was filtered, dried, and recrystallized from methanol (**SIH1**-**SIH7**, **SIH11**-**SIH13**) or methanol-ether (8:2) (**SIH8**-**SIH10**) [13].

**3-((2-Isonicotinoylhydrazono)methyl)phenyl 4-methylbenzenesulfonate (SIH1):** White solid, yield: 52% (205 mg). m.p. 209–210 °C. R_f_ (EtOAc:*n*-Hexane:MeOH = 7:2:1): 0.36. IR (ν, cm^−1^): 3323 (N-H), 1676 (C=O), 1592 (C=N), 1371, and 1152 (SO_2_ stretch). ^1^H NMR (400 MHz, DMSO-*d*_6_) *δ* 12.14 (s, 1H, -NH, D_2_O exchangeable), 8.78 (d, *J* = 4.5 Hz, 2H, Ar-H), 8.42 (s, 1H, =CH), 7.81 (d, *J*= 4.5 Hz, 2H, Ar-H), 7.75 (d, *J* = 8.0 Hz, 4H, Ar-H), 7.48 (d, *J* = 7.9 Hz, 2H, Ar-H), 7.13 (d, *J* = 8.4 Hz, 2H, Ar-H), 2.42 (s, 3H, CH_3_); ^13^C NMR (100 MHz, DMSO-*d*_6_) *δ* 162.2 (C=O), 150.8, 150.6, 147.9, 146.5, 140.8 (-C=N), 133.6, 131.6, 130.8, 129.3, 128.7, 123.1, 121.9, 21.6 (-CH_3_); HRMS: *m/z* calcd for C_20_H_17_N_3_O_4_S [M + H]^+^: 396.1018; found 396.1015.

**3-((2-Isonicotinoylhydrazono)methyl)phenyl 4-(*tert*-butyl)benzenesulfonate (SIH2):** White solid, yield: 64% (280 mg). m.p. 182–183 °C. R_f_ (EtOAc:*n*-Hexane:MeOH = 7:2:1): 0.46. IR (ν, cm^−1^): 3303 (N-H), 1663 (C=O), 1596 (C=N), 1365, and 1152 (SO_2_ stretch). ^1^H NMR (400 MHz, DMSO-*d*_6_) *δ* 12.14 (s, 1H, -NH, D_2_O exchangeable), 8.78 (d, *J* = 4.6 Hz, 2H, Ar-H), 8.43 (s, 1H, =CH), 7.82–7.80 (m, 4H, Ar-H), 7.76 (d, *J* = 8.1 Hz, 2H), 7.70 (d, *J* = 8.3 Hz, 2H), 7.15 (d, *J* = 8.2 Hz, 2H, Ar-H), 1.31 (s, 9H, CH_3_); ^13^C NMR (100 MHz, DMSO-*d*_6_) *δ* 162.2 (C=O), 158.9, 150.8, 150.6, 148.0, 140.8 (-C=N), 133.6, 131.9, 129.3, 128.6, 127.2, 123.0, 122.0, 35.6 (-C-(CH_3_)_3_), 31.1 (-CH_3_); HRMS: *m/z* calcd for C_23_H_23_N_3_O_4_S [M + H]^+^: 438.1488; found 438.1482.

**3-((2-Isonicotinoylhydrazono)methyl)phenyl 2,4,6-trimethylbenzenesulfonate (SIH3):** Yellow solid, yield: 47% (199 mg). m.p. 108–109 °C. R_f_ (EtOAc:*n*-Hexane:MeOH = 7:2:1): 0.43. IR (ν, cm^−1^): 3396 (N-H), 1661 (C=O), 1600 (C=N), 1360, and 1152 (SO_2_ stretch). ^1^H NMR (400 MHz, DMSO-*d*_6_) *δ* 12.13 (s, 1H, -NH, D_2_O exchangeable), 8.78 (d, *J* = 4.4 Hz, 2H, Ar-H), 8.41 (s, 1H, =CH), 7.80 (d, *J* = 4.6 Hz, 2H, Ar-H), 7.74 (d, *J* = 8.0 Hz, 2H, Ar-H), 7.16 (s, 2H, Ar-H), 7.09 (d, *J* = 8.1 Hz, 2H, Ar-H), 2.48 (s, 6H, CH_3_), 2.31 (s, 3H, -CH_3_); ^13^C NMR (100 MHz, DMSO-*d*_6_) *δ* 162.2 (C=O), 150.8, 150.4, 148.0, 144.9, 140.8 (-C=N), 140.3, 133.6, 132.4, 129.8, 129.3, 122.9, 121.9, 22.6 (2x-CH_3_), 21.06 (-CH_3_); HRMS: *m/z* calcd for C_22_H_21_N_3_O_4_S [M + H]^+^: 424.1331; found 424.1323.

**3-((2-Isonicotinoylhydrazono)methyl)phenyl 4-methoxybenzenesulfonate (SIH4):** White solid, yield: 71% (296 mg). m.p. 194–195 °C. R_f_ (EtOAc:*n*-Hexane:MeOH = 7:2:1): 0.33. IR (ν, cm^−1^): 3288 (N-H), 1665 (C=O), 1596 (C=N), 1369, and 1152 (SO_2_ stretch). ^1^H NMR (400 MHz, DMSO-*d*_6_) *δ* 12.14 (s, 1H, -NH, D_2_O exchangeable), 8.78 (d, *J* = 4.5 Hz, 2H, Ar-H), 8.43 (s, 1H, =CH), 7.74–7.81 (m, 6H, Ar-H), 7.17 (d, *J* = 8.2 Hz, 2H, Ar-H), 7.12 (d, *J* = 8.1 Hz, 2H, Ar-H), 3.87 (s, 3H, -OCH_3_); ^13^C NMR (100 MHz, DMSO-*d*_6_) *δ* 164.6, 162.2 (C=O), 150.8, 150.0, 148.0, 140.8 (-C=N), 133.6, 131.2, 129.3, 125.8, 123.2, 121.9, 115.5, 56.4 (-OCH_3_); HRMS: *m/z* calcd for C_20_H_17_N_3_O_5_S [M + H]^+^: 412.0967; found 412.0959.

**3-((2-Isonicotinoylhydrazono)methyl)phenyl 4-bromobenzenesulfonate (SIH5):** White solid, yield: 61% (279 mg). m.p. 217–218 °C. R_f_ (EtOAc:*n*-Hexane:MeOH = 7:2:1): 0.42. IR (ν, cm^−1^): 3306 (N-H), 1673 (C=O), 1599 (C=N), 1379, and 1150 (SO_2_ stretch). ^1^H NMR (400 MHz, DMSO-*d*_6_) *δ* 12.15 (s, 1H, -NH, D_2_O exchangeable), 8.78 (d, *J* = 4.4 Hz, 2H, Ar-H), 8.43 (s, 1H, =CH), 7.90 (d, *J* = 7.7 Hz, 2H, Ar-H), 7.81–7.77 (m, 6H, Ar-H), 7.16 (d, *J* = 7.9 Hz, 2H, Ar-H); ^13^C NMR (100 MHz, DMSO-*d*_6_) *δ* 162.2 (C=O), 150.8, 150.4, 147.9, 140.8 (-C=N), 133.9, 133.8, 133.5, 130.6, 129.9, 129.4, 123.1, 121.9, HRMS: *m/z* calcd for C_19_H_14_BrN_3_O_4_S [M + H]^+^: 459.9967; found 459.9965.

**3-((2-Isonicotinoylhydrazono)methyl)phenyl 4-chlorobenzenesulfonate (SIH6):** Yellow solid, yield: 55% (228 mg). m.p. 210–211 °C. R_f_ (EtOAc:*n*-Hexane:MeOH = 7:2:1): 0.43. IR (ν, cm^−1^): 3357 (N-H), 1665 (C=O), 1602 (C=N), 1368, and 1149 (SO_2_ stretch). ^1^H NMR (400 MHz, DMSO-*d*_6_) *δ* 12.15 (s, 1H, -NH, D_2_O exchangeable), 8.78 (d, *J* = 4.9 Hz, 2H, Ar-H), 8.43 (s, 1H, =CH), 7.89 (d, *J* = 8.1 Hz, 2H, Ar-H), 7.82–7.75 (m, 6H, Ar-H), 7.16 (d, *J* = 7.9 Hz, 2H, Ar-H); ^13^C NMR (100 MHz, DMSO-*d*_6_) *δ* 162.2 (C=O), 150.8, 150.4, 147.9, 140.8 (-C=N), 140.7, 133.9, 133.4, 130.7, 130.6, 129.4, 123.1, 121.9; HRMS: *m/z* calcd for C_19_H_14_ClN_3_O_4_S [M + H]^+^: 416.0472; found 416.0463.

**3-((2-Isonicotinoylhydrazono)methyl)phenyl 4-iodobenzenesulfonate (SIH7):** Pale yellow solid, yield: 65% (329 mg). m.p. 240–241 °C. R_f_ (EtOAc:*n*-Hexane:MeOH = 7:2:1): 0.44. IR (ν, cm^−1^): 3263 (N-H), 1660 (C=O), 1599 (C=N), 1378, and 1151 (SO_2_ stretch). ^1^H NMR (400 MHz, DMSO-*d*_6_) *δ* 12.15 (s, 1H, -NH, D_2_O exchangeable), 8.78 (d, *J* = 4.1 Hz, 2H, Ar-H), 8.43 (s, 1H, =CH), 8.07 (d, *J* = 7.2 Hz, 2H, Ar-H), 7.82–7.76 (m, 4H, Ar-H), 7.62 (d, *J* = 7.7 Hz, 2H, Ar-H), 7.16 (d, *J* = 7.9 Hz, 2H, Ar-H); ^13^C NMR (100 MHz, DMSO-*d*_6_) *δ* 162.2 (C=O), 150.8, 150.4, 147.9, 140.8 (-C=N), 139.3, 134.1, 133.9, 130.1, 129.4, 123.1, 121.9, 104.9; HRMS: *m/z* calcd for C_19_H_14_lN_3_O_4_S [M + H]^+^: 507.9828; found 507.9814.

**3-((2-Isonicotinoylhydrazono)methyl)phenyl 2,5-dichlorobenzenesulfonate (SIH8):** Yellow solid, yield: 59% (265 mg). m.p. 177–178 °C. R_f_ (EtOAc:*n*-Hexane:MeOH = 7:2:1): 0.42. IR (ν, cm^−1^): 3303 (N-H), 1667 (C=O), 1598 (C=N), 1390, and 1143 (SO_2_ stretch). ^1^H NMR (400 MHz, DMSO-*d*_6_) *δ* 12.21 (s, 1H, -NH, D_2_O exchangeable), 8.78 (d, *J* = 4.8 Hz, 2H, Ar-H), 8.46 (s, 1H, =CH), 7.93–7.78 (m, 7H, Ar-H), 7.25 (d, *J* = 8.3 Hz, 2H, Ar-H); ^13^C NMR (100 MHz, DMSO-*d*_6_) *δ* 162.2 (C=O), 150.8, 150.1, 147.9, 140.7 (-C=N), 136.9, 134.8, 134.1, 133.2, 131.7, 131.2, 129.6, 122.7, 122.0, 121.5; HRMS: *m/z* calcd for C_19_H_13_Cl_2_N_3_O_4_S [M + H]^+^: 450.0082; found 450.0076.

**3-((2-Isonicotinoylhydrazono)methyl)phenyl 4-nitrobenzenesulfonate (SIH9):** White solid, yield: 77% (328 mg). m.p. 211–212 °C. R_f_ (EtOAc:*n*-Hexane:MeOH = 7:2:1): 0.51. IR (ν, cm^−1^): 3248 (N-H), 1663 (C=O), 1602 (C=N), 1379, and 1147 (SO_2_ stretch). ^1^H NMR (400 MHz, DMSO-*d*_6_) *δ* 12.17 (s, 1H, -NH, D_2_O exchangeable), 8.78 (d, *J* = 4.5 Hz, 2H, Ar-H), 8.46 (d, *J* = 8.3 Hz, 2H, Ar-H), 8.43 (s, 1H, =CH), 8.17 (d, *J* = 8.1 Hz, 2H, Ar-H), 7.81–7.77 (m, 4H, Ar-H), 7.18 (d, *J* = 8.0 Hz, 2H, Ar-H); ^13^C NMR (100 MHz, DMSO-*d*_6_) *δ* 162.4 (C=O), 151.6, 150.8, 150.2, 148.0, 140.7 (-C=N), 139.7, 133.9, 130.5, 129.6, 125.5, 123.1, 122.0; HRMS: *m/z* calcd for C_19_H_14_N_4_O_6_S [M + H]^+^: 427.0707; found 427.0685.

**3-((2-Isonicotinoylhydrazono)methyl)phenyl 3-nitrobenzenesulfonate (SIH10):** White solid, yield: 64% (264 mg). m.p. 203–204 °C. R_f_ (EtOAc:*n*-Hexane:MeOH = 7:2:1): 0.58. IR (ν, cm^−1^): 3267 (N-H), 1651 (C=O), 1607 (C=N), 1379, and 1148 (SO_2_ stretch). ^1^H NMR (400 MHz, DMSO-*d*_6_) *δ* 12.17 (s, 1H, -NH, D_2_O exchangeable), 8.78 (d, *J* = 4.8 Hz, 2H, Ar-H), 8.66 (d, *J* = 8.0 Hz, 1H, Ar-H), 8.50 (s, 1H, Ar-H), 8.43 (s, 1H, =CH), 8.29 (d, *J* = 8.1 Hz, 1H, Ar-H), 7.97 (t, *J* = 8.0 Hz, 1H, Ar-H), 7.81–7.76 (m, 4H, Ar-H), 7.22 (d, *J* = 8.1 Hz, 2H, Ar-H); ^13^C NMR (100 MHz, DMSO-*d*_6_) *δ* 162.2 (C=O), 150.8, 150.2, 148.6, 147.9, 140.8 (-C=N), 135.8, 134.6, 134.1, 132.5, 130.2, 129.5, 123.5, 123.3, 122.0; HRMS: *m/z* calcd for C_19_H_14_N_4_O_6_S [M + H]^+^: 427.0707; found 427.0682.

**3-((2-Isonicotinoylhydrazono)methyl)phenyl 4-acetamidobenzenesulfonate (SIH11):** White solid, yield: 68% (298 mg). m.p. 193–194 °C. R_f_ (EtOAc:*n*-Hexane:MeOH = 7:2:1): 0.35. IR (ν, cm^−1^): 3257 (N-H), 1670 (C=O), 1597 (C=N), 1352, and 1147 (SO_2_ stretch). ^1^H NMR (400 MHz, DMSO-*d*_6_) *δ* 12.13 (s, 1H, -NH, D_2_O exchangeable) 10.50 (s, 1H, -NH), 8.77 (d, *J* = 3.8 Hz, 2H, Ar-H), 8.42 (s, 1H, =CH), 7.82–7.74 (m, 8H, Ar-H), 7.12 (d, *J* = 8.0 Hz, 2H, Ar-H), 2.10 (s, 3H, -CH_3_); ^13^C NMR (100 MHz, DMSO-*d*_6_) *δ* 169.9 (C=O), 162.2 (C=O), 150.8, 150.6, 148.1, 145.5, 140.8 (-C=N), 133.6, 130.2, 129.3, 127.5, 123.2, 121.9, 119.2, 24.7 (-CH_3_); HRMS: *m/z* calcd for C_21_H_18_N_4_O_5_S [M + H]^+^: 439.1076; found 439.1066.

**3-((2-Isonicotinoylhydrazono)methyl)phenyl [1,1′-biphenyl]-4-sulfonate (SIH12):** Orange solid, yield: 53% (242 mg).. m.p. 118–119 °C. R_f_ (EtOAc:*n*-Hexane:MeOH = 7:2:1): 0.44. IR (ν, cm^−1^): 3382 (N-H), 1660 (C=O), 1596 (C=N), 1372, and 1151 (SO_2_ stretch). ^1^H NMR (400 MHz, DMSO-*d*_6_) *δ* 12.16 (s, 1H, -NH, D_2_O exchangeable), 8.78 (d, *J* = 4.7 Hz, 2H, Ar-H), 8.43 (s, 1H, =CH), 8.00–7.94 (m, 4H, Ar-H), 7.81–7.77 (m, 5H, Ar-H), 7.55–7.45 (m, 4H, Ar-H), 7.19 (d, *J* = 8.3 Hz, 2H, Ar-H); ^13^C NMR (100 MHz, DMSO-*d*_6_) *δ* 162.2 (C=O), 150.8, 150.5, 148.0, 146.8, 140.8 (-C=N), 138.2, 133.7, 133.2, 129.7, 129.6, 129.4, 128.3, 127.7, 127.5, 123.6, 121.9; HRMS: *m/z* calcd for C_25_H_19_N_3_O_4_S [M + H]^+^: 458.1175; found 458.1166.

**3-((2-Isonicotinoylhydrazono)methyl)phenyl quinoline-8-sulfonate (SIH13):** White solid, yield: 72% (327 mg). m.p. 231–232 °C. R_f_ (EtOAc:*n*-Hexane:MeOH = 7:2:1): 0.22. IR (ν, cm^−1^): 3294 (N-H), 1670 (C=O), 1598 (C=N), 1359, and 1142 (SO_2_ stretch). ^1^H NMR (400 MHz, DMSO-*d*_6_) *δ* 12.11 (s, 1H, -NH, D_2_O exchangeable), 9.23 (s, 1H, Ar-H), 8.77 (d, *J* = 4.2 Hz, 2H, Ar-H), 8.64 (d, *J* = 8.2 Hz, 1H, Ar-H), 8.47 (d, *J* = 8.1 Hz, 1H, Ar-H), 8.39 (d, *J* = 4.9 Hz, 1H, Ar-H), 8.37 (s, 1H, =CH), 7.82–7.77 (m, 4H, Ar-H), 7.68 (d, *J* = 8.2 Hz, 2H, Ar-H), 7.10 (d, *J* = 8.2 Hz, 2H, Ar-H); ^13^C NMR (100 MHz, DMSO-*d*_6_) *δ* 162.1 (C=O), 152.9, 150.9, 150.8, 148.0, 143.5, 140.8 (-C=N), 137.7, 136.9, 134.5, 133.4, 131.7, 129.2, 126.2, 123.6, 122.9, 122.5, 121.9; HRMS: *m/z* calcd for C_22_H_16_N_4_O_4_S [M+Na]^+^: 455.0790; found 455.0788.

### 3.2. Microplate Alamar Blue Assay (MABA) Protocol

*Mycobacterium tuberculosis* H37Rv (*Mtb*) obtained from the American Type Culture Collection (ATCC) and drug-resistant clinical isolates from Oslo University Hospital were streaked onto 7H10+OADC agar plates and incubated at 37 °C. In OADC-enriched liquid Middlebrook 7H9 medium, pure colonies from agar plates were grown to the middle of the log phase. Subsequently, *Mtb* cultures were exponentially grown and inoculated into Middlebrook 7H9 medium on 96-well plates at progressively higher concentrations of the testing chemicals, with approximately 4 × 105 CFU/mL in 200 µL of each well. Before receiving 32.5 µL of a resazurin-tween mixture (8:5 ratio of 0.6 mM Resazurin in 1× PBS to 20% Tween 80), plates were incubated at 37 °C for one week. The production of fluorescent resorufin assists in determining the minimum inhibitory concentration (MIC) of the compounds tested [13].

### 3.3. Cytotoxicity Protocol

The MTT (3-(4,5-dimethylthiazol-2-yl)-2,5-diphenyltetrazolium bromide) test was used to assess the cytotoxicity of the compounds against the cell lines Human Embryonic Kidney cells (HEK 293), Normal Lung Fibroblasts (IMR-90), and Human non-tumorigenic lung epithelial cells (BEAS-2B). All cell lines were obtained from ATCC. When testing compounds **SIH1**–**SIH13**, they were added by the use of double dilution across 6 dosages ranging from 100 µM to 3.12 µM to a sterile 96 well microtiter plate containing 1 × 10^4^ cells/well and cultured for 48 h at 37 °C with 5% CO_2_. The medium was removed, and 10 µL of MTT reagent (5 mg/mL) was added to the wells and incubated for another 3 h. The MTT reagent was then removed, and 100 µL of DMSO was added into each well. DMSO dissolves the formazan crystals that have formed in the wells. The absorbance was measured at 580 nm against a blank using the Thermo Scientific varioskan lux microplate reader, and the IC_50_ values were calculated by using GraphPad Prism [36,37].

### 3.4. Stability Studies

The T80+ series of UV/Vis Spectrophotometers (PG Instruments Ltd.) were used for stability assessment studies. The stability experiments were performed at 25 °C with wavelength ranges of 260–800 nm. DMSO and EtOH were purchased as reagent grade and used without any purification. Samples with a concentration of 6.0 × 10^–5^ M were prepared in DMSO and EtOH-water. The change in λ_max_ values throughout the 24 h in the selected solvents was monitored to assess the stability of the hydrazone moieties.

### 3.5. InhA Inhibition

#### 3.5.1. Production and Purification of InhA

InhA sequence (UniProtKB: P9WGR1) has been optimized for *Escherichia coli* BL21 DE3 strain with GenSmart™ Codon Optimization (GenScript) and inserted in pET15b plasmid using the NdeI and BamHI restriction sites. BL21 (DE3) strains have been transformed with pET15b-InhA plasmid. A colony from this transformation was grown in LB medium containing 100 mg/mL ampicillin at 37 °C 180 rpm overnight. Then, 10 mL of this solution was added to 990 mL of LB medium containing ampicillin (100 mg/mL) and incubated at 37 °C 150 rpm until an optical density (OD) at 600 nm of 0.6–0.8 was reached. Once the OD600 was reached, 1 mL of 1 M isopropyl β-D-1-thiogalactopyranoside (IPTG) was added and the solution was incubated at 30 °C for 4 h at 150 rpm. Bacteria cells were collected by centrifugation at 6000× *g* for 30 min at 4 °C. Cells from pellets were solubilized with buffer (20 mM Tris-HCl, 300 mM NaCl, 10 mM imidazole, pH 7.9) and lysed using an Emulsiflex-C5 cell disruptor (Avestin). The lysate was centrifuged at 25,000× *g* for 30 min at 4 °C, the supernatant was filtered off with a 0.2 µm filter and injected into a nickel-chelated Hi-Trap HP 1 mL (Cytiva) column previously equilibrated with washing buffer (20 mM Tris-HCl, 300 mM NaCl, 10 mM imidazole, pH 7.9). After washing out the unbound proteins, bound His6-InhA was eluted using a buffer with higher imidazole concentration (20 mM Tris-HCl, 300 mM NaCl, 300 mM imidazole, pH 7.9). Fractions corresponding to His6-InhA were pooled together and concentrated using centrifugal concentrator (Vivaspin 10,000 MWCO). Then, the concentrated protein solution was centrifuged at 10,000× *g* for 10 min at 4 °C to remove aggregates and injected into a SepFast 16/60 6–600 kDa pg column (BioToolomics) previously equilibrated with buffer (30 mM PIPES, 150 mM NaCl, pH 6.8). Fractions corresponding to His6-InhA were pooled together then aliquots of 200 µL at 5 µM were prepared followed by flash freezing in liquid nitrogen. Frozen His6-InhA proteins were stored at −80 °C until usage.

#### 3.5.2. Enzymatic Assay

InhA activity assays were monitored by following NADH concentration at 340 nm with a Cary 300 UV-vis (Agilent) spectrophotometer. As described previously [38], 2-trans-dodecenoyl-CoA (DDCoA) was used as an analogue of the substrate for this reaction. Briefly, to 840 µL of buffer (30 mM PIPES, 150 mM NaCl, pH 6.8) in 1 mL cuvette, inhibitor in DMSO at constant 5% *v*/*v* of DMSO (50 µL), 50 µL DDCoA of 1 mM stock in buffer and 50 µL of NADH (Alfa Aesar) of 5 mM stock in buffer were added. After a short period of 2 min pre-incubation at 25 °C, the reaction was started by the addition of 10 µL His6-InhA from a 5 µM stock solution prepared as explained before. Rate of NADH oxidation during His6-InhA activity is monitored in the presence or in the absence of inhibitor. The inhibitory activity of tested molecules is expressed as the percentage inhibition of His6-InhA activity (with respect to control reaction) without inhibitor, as shown below. All activity assays were performed in duplicate or triplicate.

$$\mathrm{inhibition}\;\%=\frac{\mathrm{Reference}\;\mathrm{slope}-\mathrm{Compound}\;\mathrm{slope}}{\mathrm{Reference}\;\mathrm{slope}}\;\times \;100$$

### 3.6. In Silico Studies

#### 3.6.1. Molecular Docking

The 3D crystal structure of *Mycobacterium tuberculosis* enoyl-ACP reductase (InhA) with its native inhibitor GEQ, (4-(9*H*-fluoren-9-yl)piperazin-1-yl)(1*H*-indol-5-yl)methanone, was downloaded from Protein Data Bank under the PDB Code 1P44 [39]. The chemical structure of **SIH7** showing the best inhibition value against InhA among the tested compounds was sketched and MMFF94 force field in LigandScout 4.4 was used to minimize the energy of the 3D structure [40]. **SIH7** was docked into the binding pocket of InhA with default parameters of AutoDock [41], implemented in LigandScout 4.4. The obtained modes were ranked based on their estimated binding energies and the figures of the most plausible binding pose were generated using LigandScout and Maestro [42].

#### 3.6.2. Prediction of Drug-Likeness

The chemical structures of all compounds were drawn and their significant parameters were predicted by using SwissADME [43].

## 4. Conclusions

The rapid emergence and global spread of drug-resistant strains of *Mtb* have once again made TB an urgent concern to human health around the world. Particularly, the resistance developed against INH, as the frontline drug of TB treatment, has appeared as a significant handicap to controlling the global prevalence of the disease. In this study, we focused on the chemical modifications of inexpensive INH to provide a new route towards the antitubercular drug development process. Accordingly, we synthesized new INH derivatives by linking this scaffold to sulfonate esters with a hydrazone functionality (**SIH1–SIH13**). We established the stability of the compounds both in DMSO and water to eliminate the possibility that the antitubercular effect was due to INH released by hydrolyzation during biological experiments. The antitubercular and toxicity data obtained from MABA and MTT assays respectively confirmed the potential of the compounds in TB treatment. Testing the inhibitory activity of **SIH1–SIH13** against InhA, the primary target of INH, showed that our compounds can be hosted within the binding pocket of the enzyme. However, the antimycobacterial activity was not mainly due to InhA inhibition. Calculating some critical physicochemical descriptors of the compounds confirmed their drug-likeness and emphasized the importance of the increased lipophilicity in the biological profile of **SIH1–SIH13** as compared to INH. Altogether, our study presented a new approach for modifying INH to overcome the challenging drug resistance problem in TB treatment.

## Figures and Tables

**Table 1:** MIC values against *Mtb* H37Rv and INH-resistant clinical isolates, and cytotoxicity data of **SIH1–SIH13**.

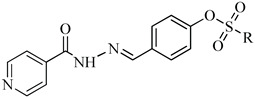							
		MIC (μM)			Toxicity * IC_50_ (µM)		
Compound	R	H37Rv	*inhA* ^+^	*katG* ^+^	HEK293	IMR-90	BEAS-2B
**SIH1**	4-methylphenyl	0.31	1.56	50	57.21	>100	>100
**SIH2**	4-(*tert*-butyl)phenyl	0.62	1.56	25	>100	89.50	>100
**SIH3**	2,4,6-trimethylphenyl	0.62	6.25	50	100	>100	>100
**SIH4**	4-methoxyphenyl	0.31	1.56	6.25	40.76	>100	>100
**SIH5**	4-bromophenyl	0.62	3.12	25	100	90.32	>100
**SIH6**	4-chlorophenyl	0.62	3.12	>100	>100	>100	>100
**SIH7**	4-iodophenyl	0.62	6.25	>100	>100	100	>100
**SIH8**	2,5-dichlorophenyl	0.62	3.12	12.5	>100	100	>100
**SIH9**	4-nitrophenyl	0.62	3.12	25	100	>100	>100
**SIH10**	3-nitrophenyl	0.62	3.12	12.5	>100	>100	>100
**SIH11**	4-acetamidophenyl	0.62	3.12	12.5	>100	>100	>100
**SIH12**	1,1′-biphenyl	0.31	3.12	12.5	52.40	57.7	>100
**SIH13**	quinoline-8-yl	0.31	3.12	12.5	58.34	100	>100
**Isoniazid**		0.31	1.56	6.25	NT	NT	NT

NT: Not Tested, * experiments were performed in triplicate.

**Table 2:** Inhibitory results for **SIH1–SIH13** against InhA enzyme at 50 µM concentration.

Compound	% Inhibition	Compound	% Inhibition
**SIH1**	12	**SIH8**	NI
**SIH2**	25	**SIH9**	8
**SIH3**	NI	**SIH10**	17
**SIH4**	5	**SIH11**	10
**SIH5**	33	**SIH12**	31
**SIH6**	23	**SIH13**	22
**SIH7**	41	**TCL**	98

NI: No inhibition.

**Table 3:** Calculated molecular properties of **SIH1–SIH13**.

Compound	MW ^a^	LogP ^b^	HBD ^c^	HBA ^d^	NRB ^e^	TPSA ^f^	Lipinski’s Violation
**SIH1**	395.43	2.95	1	6	7	106.10	0
**SIH2**	437.51	3.87	1	6	8	106.10	0
**SIH3**	423.48	3.74	1	6	7	106.10	0
**SIH4**	411.43	2.70	1	7	8	115.33	0
**SIH5**	460.30	3.22	1	6	7	106.10	0
**SIH6**	415.85	3.15	1	6	7	106.10	0
**SIH7**	507.30	3.25	1	6	7	106.10	1
**SIH8**	450.30	3.61	1	6	7	106.10	0
**SIH9**	426.40	1.98	1	8	8	151.92	0
**SIH10**	426.40	2.04	1	8	8	151.92	0
**SIH11**	438.46	2.25	2	7	9	135.20	0
**SIH12**	457.50	3.94	1	6	8	106.10	0
**SIH13**	432.45	2.97	1	7	7	118.99	0

^a^ MW: molecular weight. ^b^ LogP: logarithm of *n*-octanol-water partition coefficient. ^c^ HBA: number of hydrogen bond acceptors. ^d^ HBD: number of hydrogen bond donors. ^e^ NRB: number of rotatable bonds. ^f^ TPSA: topological polar surface area.

## Data Availability

Data are contained within the article or Supplementary Materials.

## Supplementary Materials

The following supporting information can be downloaded at: https://www.mdpi.com/article/10.3390/ph15101301/s1, ^1^H NMR, ^13^C NMR, FTIR, and HRMS spectra of all synthesized compounds, InhA optimised sequence, UV/Vis spectra of **SIH1**, **SIH4**, and **SIH12** in DMSO and EtOH-water, and molecular docking figures and discussion are provided.
