# Supplementary material for: Isoniazid Linked to Sulfonate Esters via Hydrazone Functionality: Design, Synthesis, and Evaluation of Antitubercular Activity
Source: Pharmaceuticals (Basel). 2022 Oct 21;15(10):1301. doi: 10.3390/ph15101301 (PMC9609273; doi:10.3390/ph15101301)
Supplement: Supplementary file 1 [file pharmaceuticals-15-01301-s001.zip › pharmaceuticals-1935217-supplementary.pdf]

Supplementary Material

Isoniazid linked to sulfonate esters via hydrazone functionality: Design, synthesis, and evaluation of antitubercular activity

Ebru Koçak Aslan[a], M. İhsan Han[b], Vagolu Siva Krishna[c], Rasoul Tamhaev[d,e], Cagatay Dengiz[f], Şengül Dilem Doğan[g], Christian Lherbet[d], Lionel Mourey[e], Tone Tønjum[c,h], Miyase Gözde Gündüz[a]*

[a]Department of Pharmaceutical Chemistry, Faculty of Pharmacy, Hacettepe University, Sıhhiye, 06100, Ankara, Turkey

[b]Department of Pharmaceutical Chemistry, Faculty of Pharmacy, Erciyes University, 38039, Kayseri, Turkey

[c]Unit for Genome Dynamics, Department of Microbiology, University of Oslo, Oslo, Norway

[d] SPCMIB, UMR5068, CNRS, Université de Toulouse, UPS, Toulouse, France.

[e]Institut de Pharmacologie et de Biologie Structurale, IPBS, Université de Toulouse, CNRS, UPS, Toulouse, France.

[f]Department of Chemistry, Middle East Technical University, 06800, Ankara, Turkey

[g]Department of Basic Sciences, Faculty of Pharmacy, Erciyes University, 38039, Kayseri, Turkey

[h]Unit for Genome Dynamics, Department of Microbiology, Oslo University Hospital, Oslo, Norway

***Corresponding author**

Dr. Miyase Gözde GÜNDÜZ

Address: Hacettepe University

Faculty of Pharmacy

Department of Pharmaceutical Chemistry

06100 Sıhhiye

Ankara, TURKEY

E-mail address: miyasegunduz@yahoo.com

**Table of Contents**	**Page**
^{1}H NMR, ^{13}C NMR, HRMS, and FTIR Spectra of **SIH1-SIH13**	**3-28**
InhA optimised sequence	**29**
Stability assessment of **SIH1**, **SIH4**, and **SIH12** in DMSO and EtOH-water	**30**
Molecular docking studies	**31-32**

^{1}H NMR, ^{13}C NMR, HRMS and FTIR spectra of **SIH1**

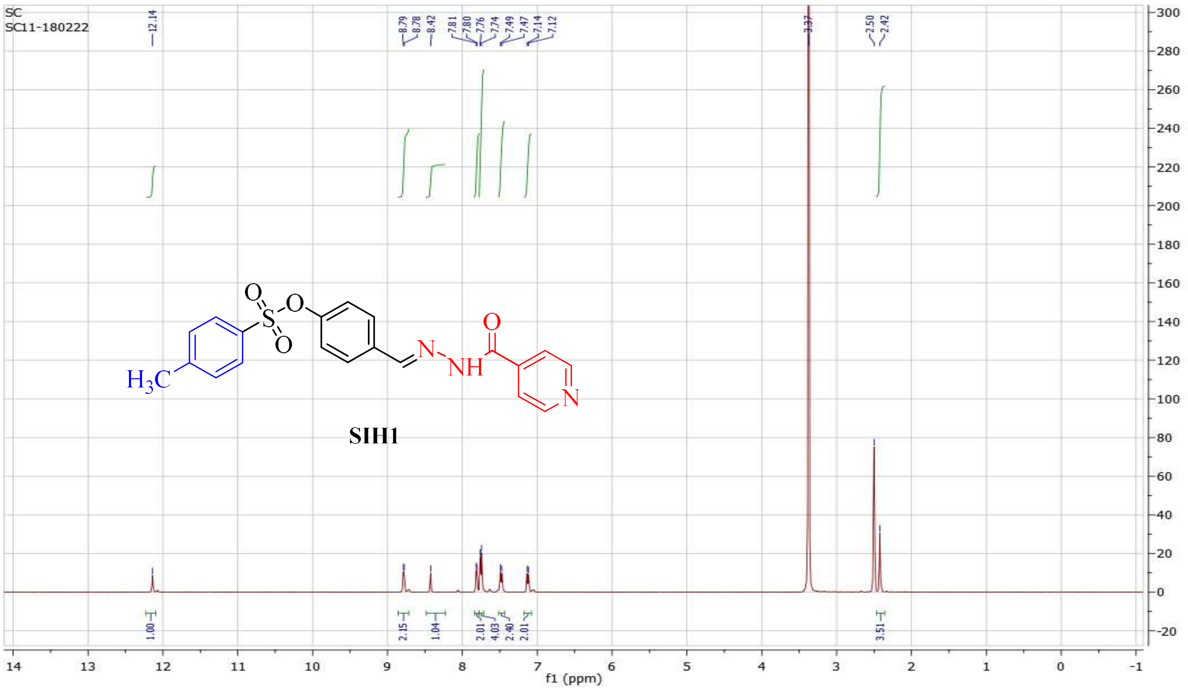

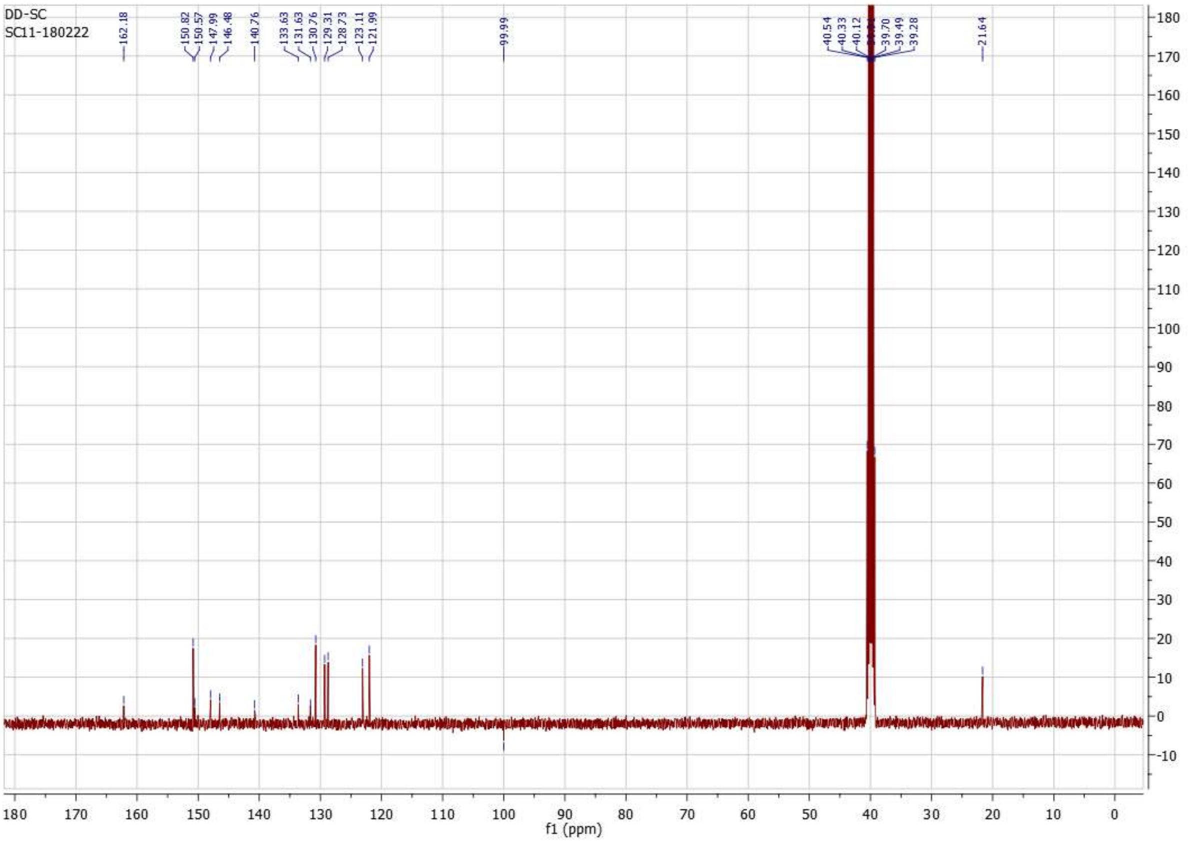

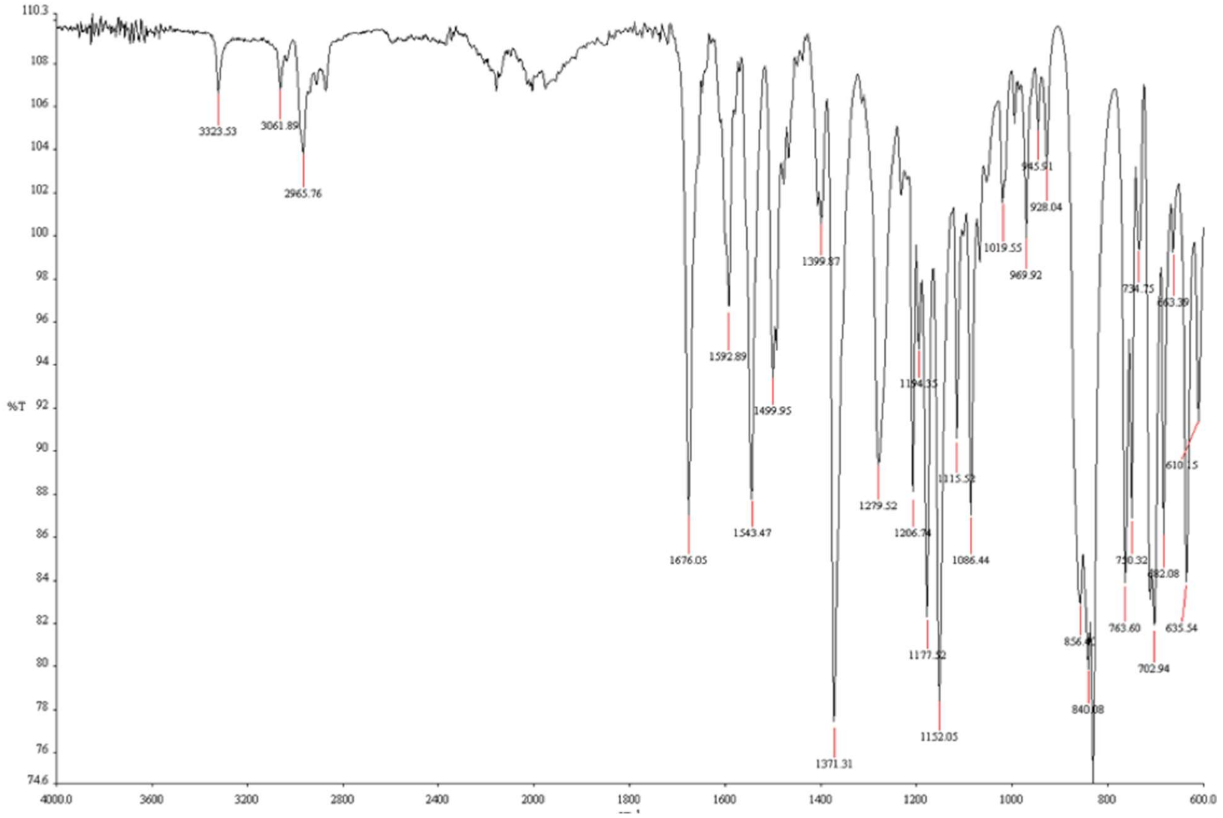

User Spectra

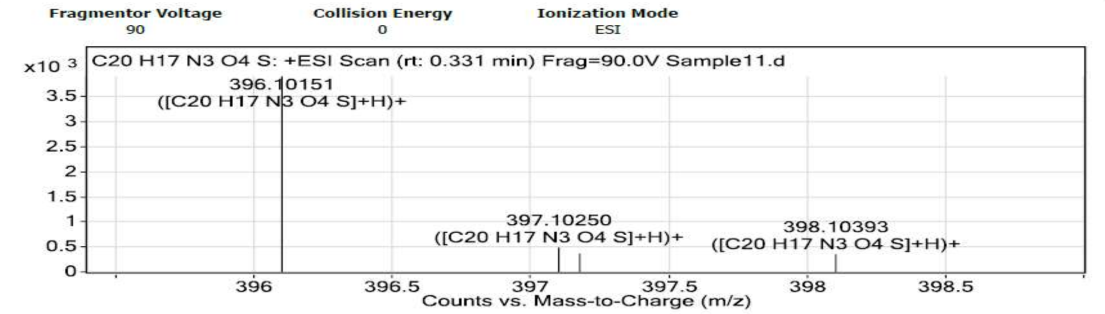

^{1}H NMR, ^{13}C NMR, HRMS and FTIR spectra of **SIH2**

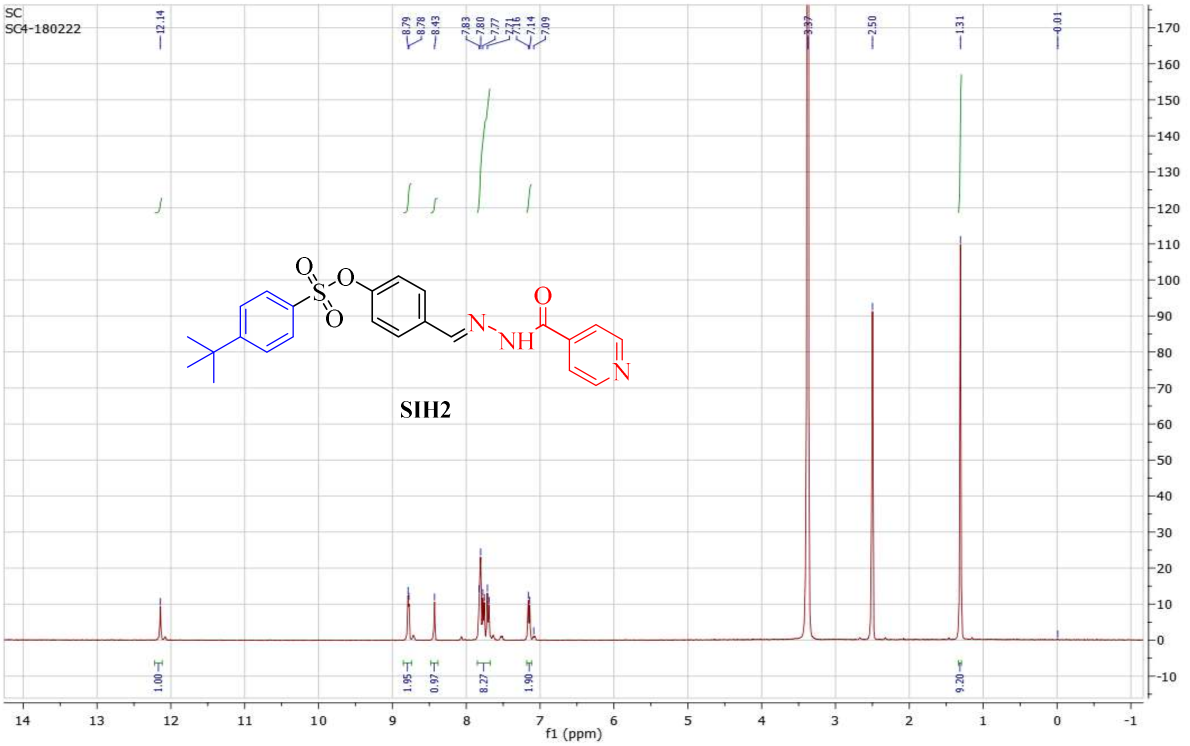

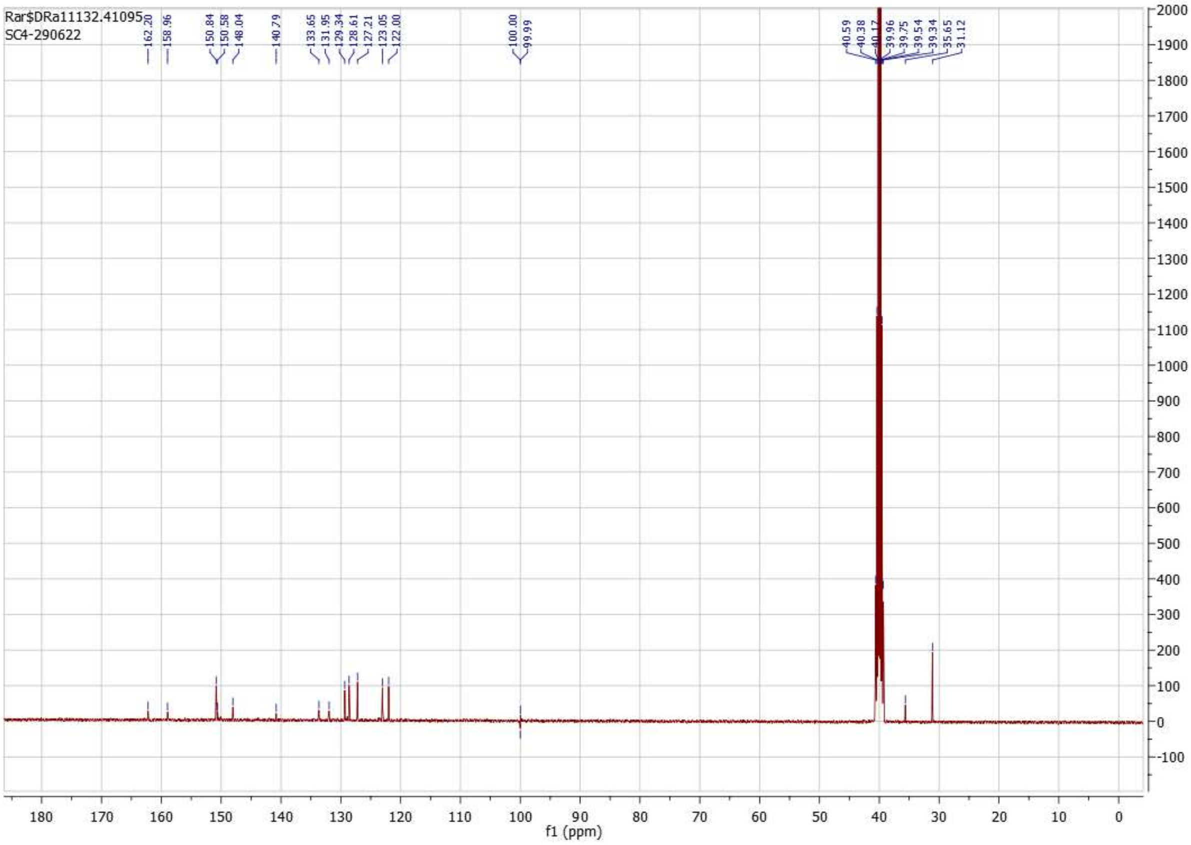

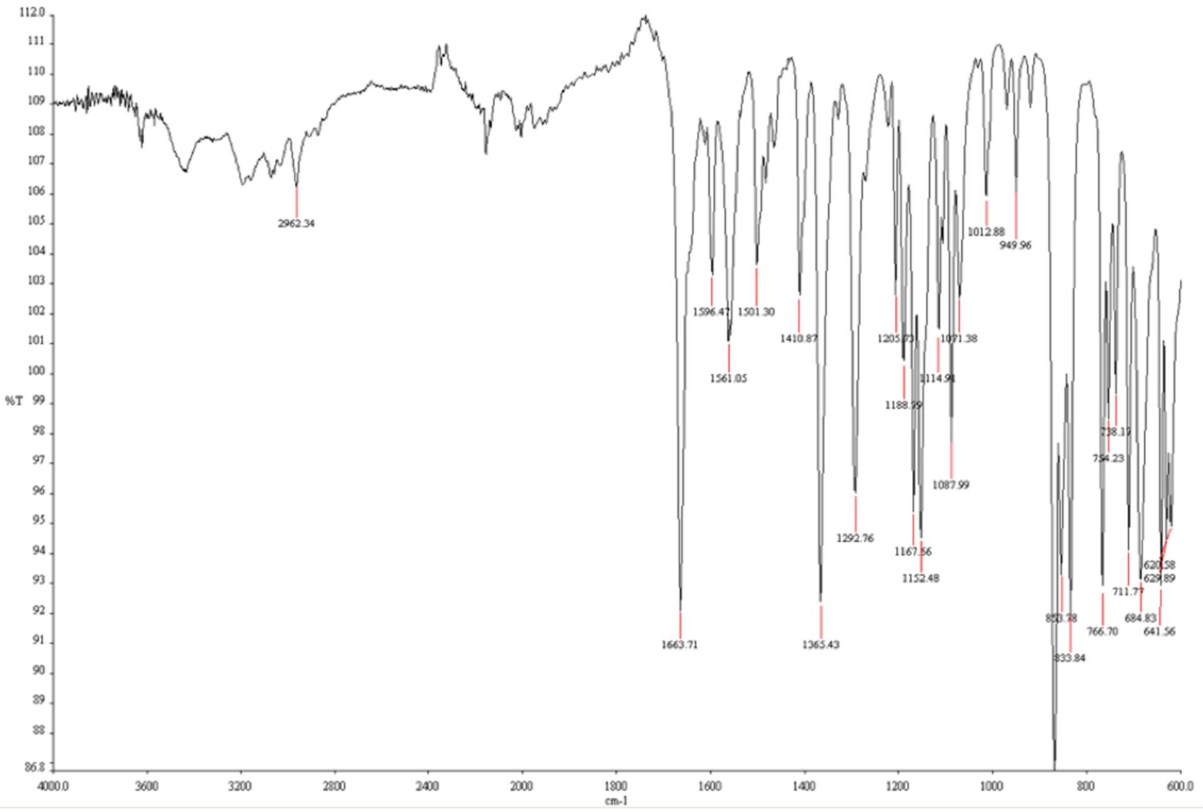

%T
cm-1
2962.34
1663.71
1365.43
1292.76
1596.47
1561.05
1501.30
1410.87
1205.73
1188.59
1167.56
1152.48
1114.91
1087.99
1071.38
1012.88
949.96
853.78
833.84
766.70
754.23
738.17
711.77
684.83
641.56
629.89
620.58

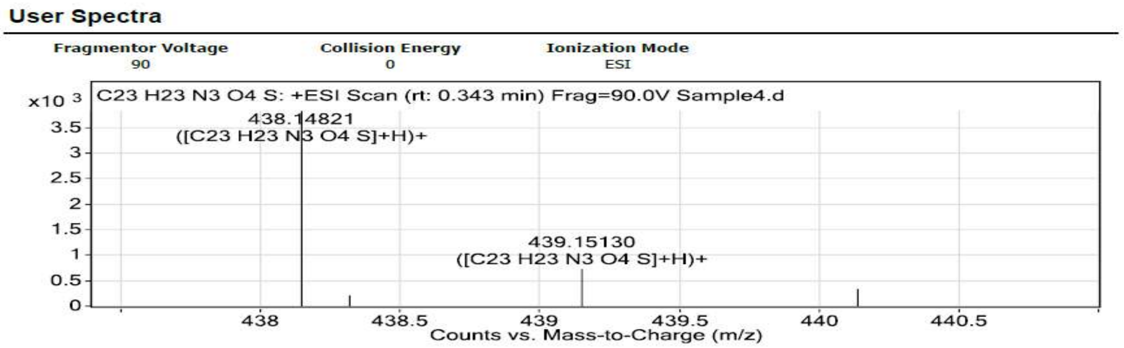

User Spectra
Fragmentor Voltage
90
Collision Energy
0
Ionization Mode
ESI
C23 H23 N3 O4 S: +ESI Scan (rt: 0.343 min) Frag=90.0V Sample4.d
x10 3
438.14821
([C23 H23 N3 O4 S]+H)+
439.15130
([C23 H23 N3 O4 S]+H)+
Counts vs. Mass-to-Charge (m/z)

^{1}H NMR, ^{13}C NMR, HRMS and FTIR spectra of **SIH3**

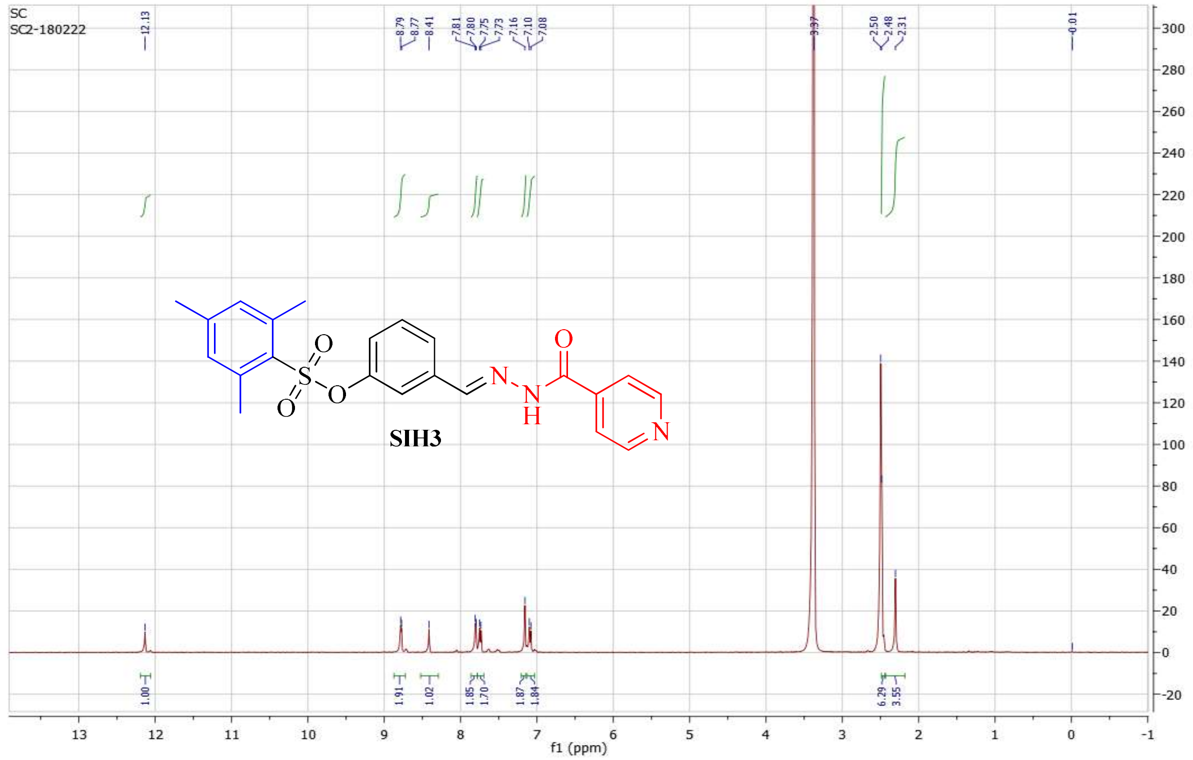

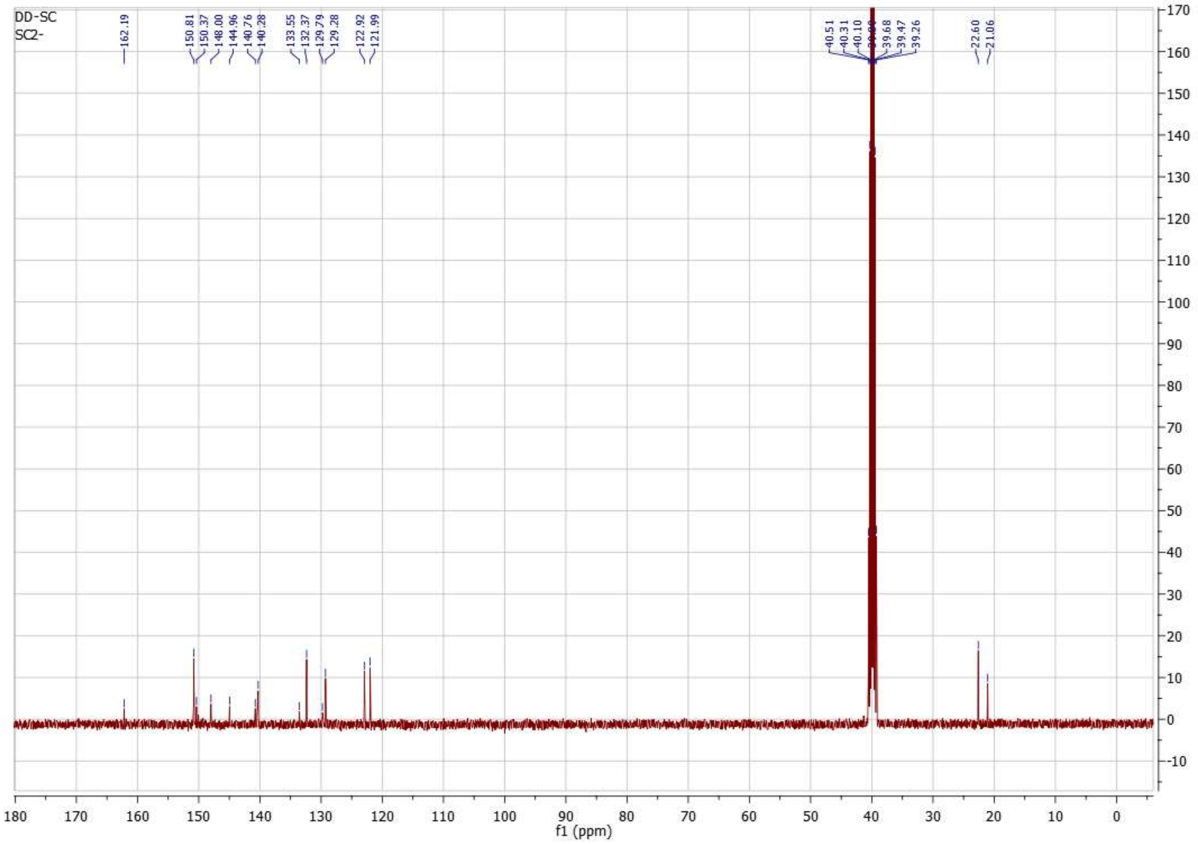

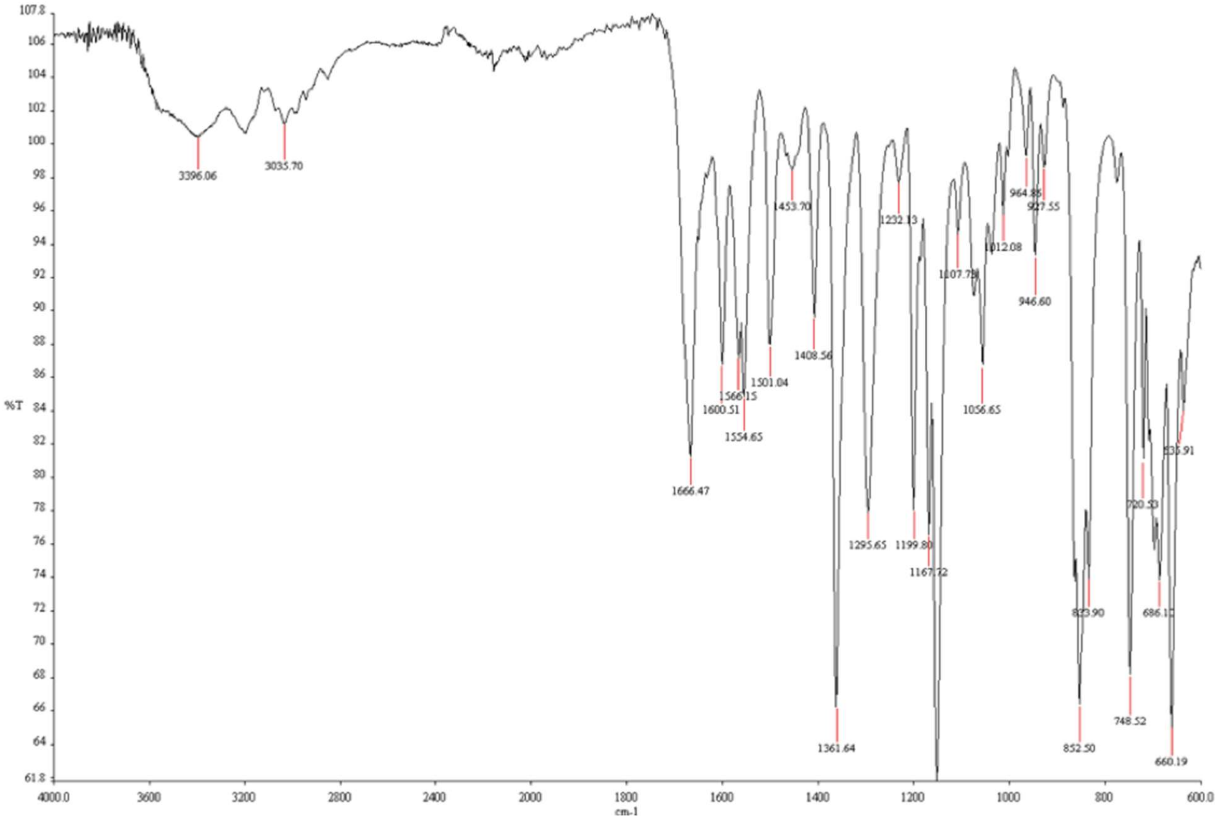

%T
cm-1
3396.06
3035.70
1666.47
1600.51
1566.15
1554.65
1501.04
1453.70
1408.56
1361.64
1295.65
1232.13
1199.80
1056.65
1012.08
946.60
927.55
852.50
823.90
748.52
720.53
686.12
660.19

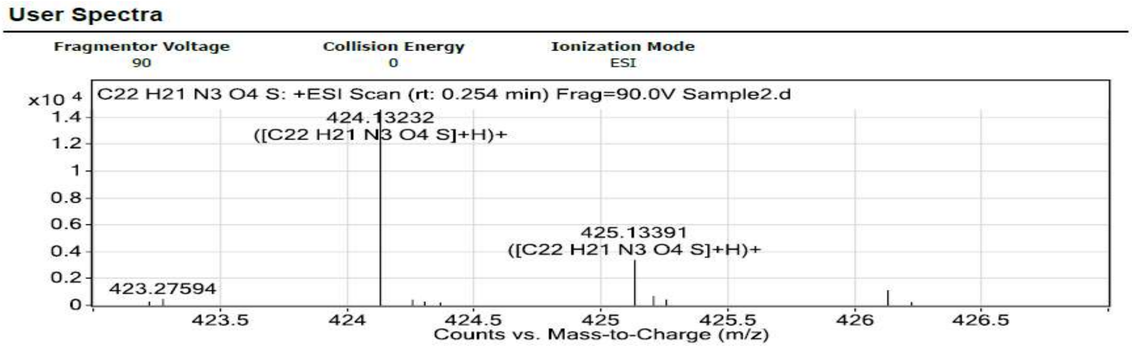

User Spectra
Fragmentor Voltage
90
Collision Energy
0
Ionization Mode
ESI
x10 4
C22 H21 N3 O4 S: +ESI Scan (rt: 0.254 min) Frag=90.0V Sample2.d
424.13232
([C22 H21 N3 O4 S]+H)+
425.13391
([C22 H21 N3 O4 S]+H)+
423.27594
Counts vs. Mass-to-Charge (m/z)

^{1}H NMR, ^{13}C NMR, HRMS and FTIR spectra of **SIH4**

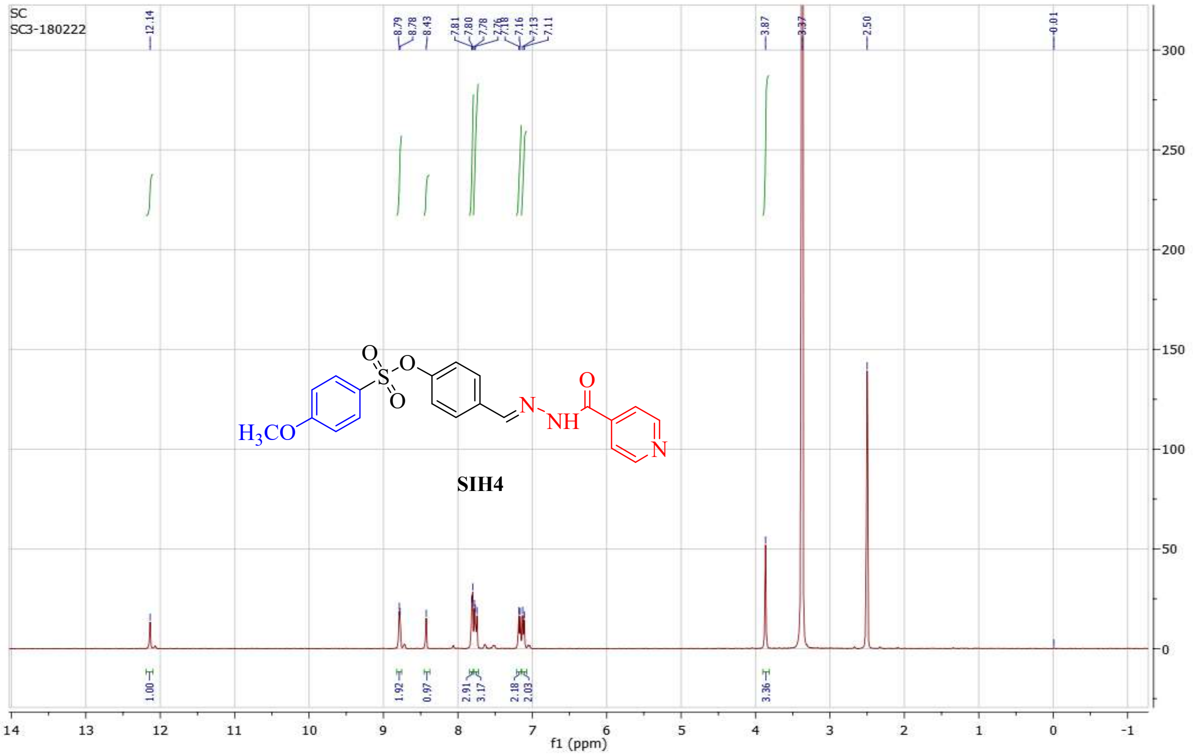

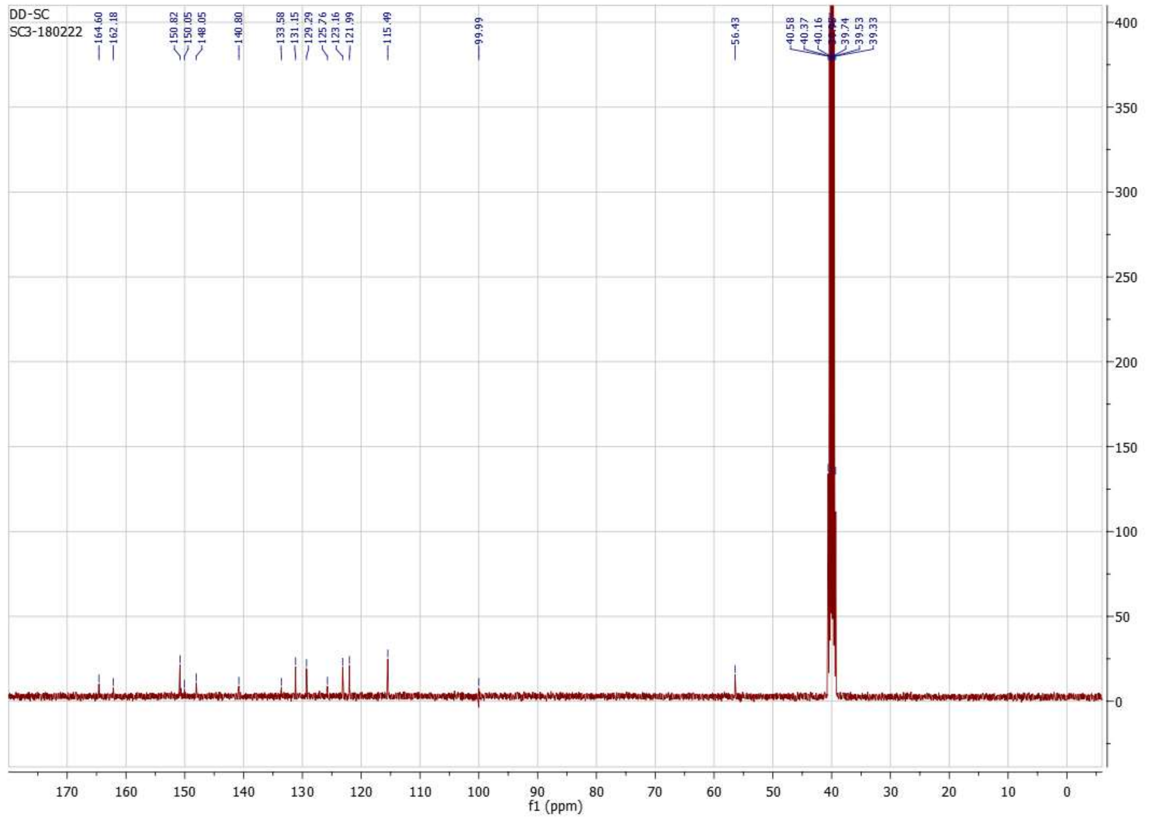

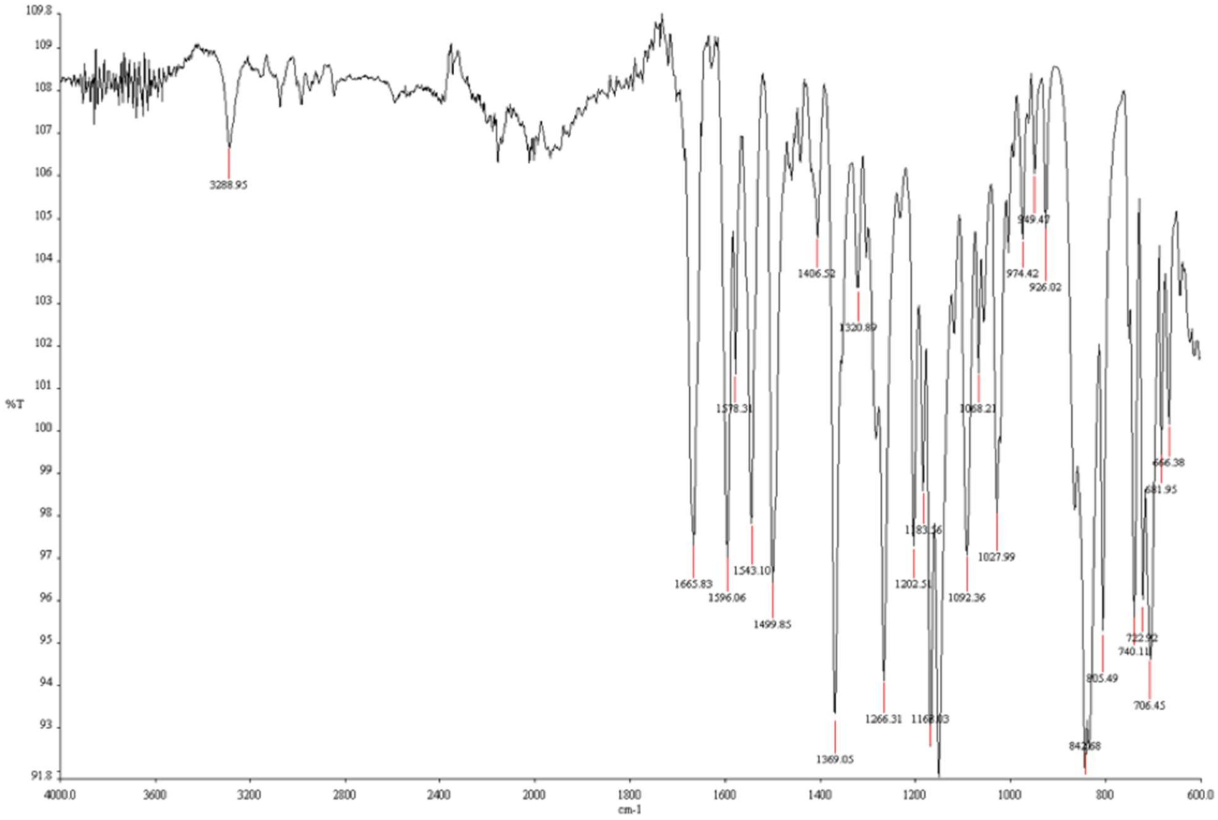
%T
cm-1
3288.95
1665.83
1596.06
1578.31
1543.10
1499.85
1406.52
1369.05
1320.89
1266.31
1202.51
1092.36
1068.21
1027.99
974.42
949.47
926.02
805.49
740.11
722.92
706.45
681.95
666.38

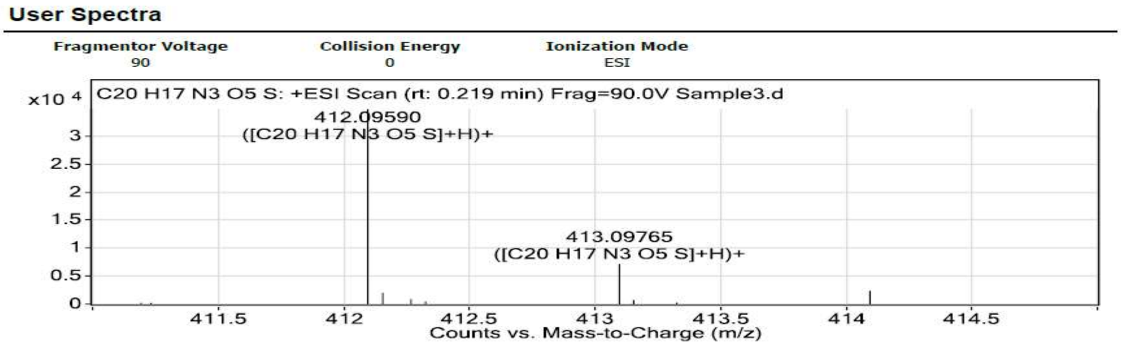
User Spectra
Fragmentor Voltage
90
Collision Energy
0
Ionization Mode
ESI
C20 H17 N3 O5 S: +ESI Scan (rt: 0.219 min) Frag=90.0V Sample3.d
x10 4
412.09590
([C20 H17 N3 O5 S]+H)+
413.09765
([C20 H17 N3 O5 S]+H)+
Counts vs. Mass-to-Charge (m/z)

^{1}H NMR, ^{13}C NMR, HRMS and FTIR spectra of **SIH5**

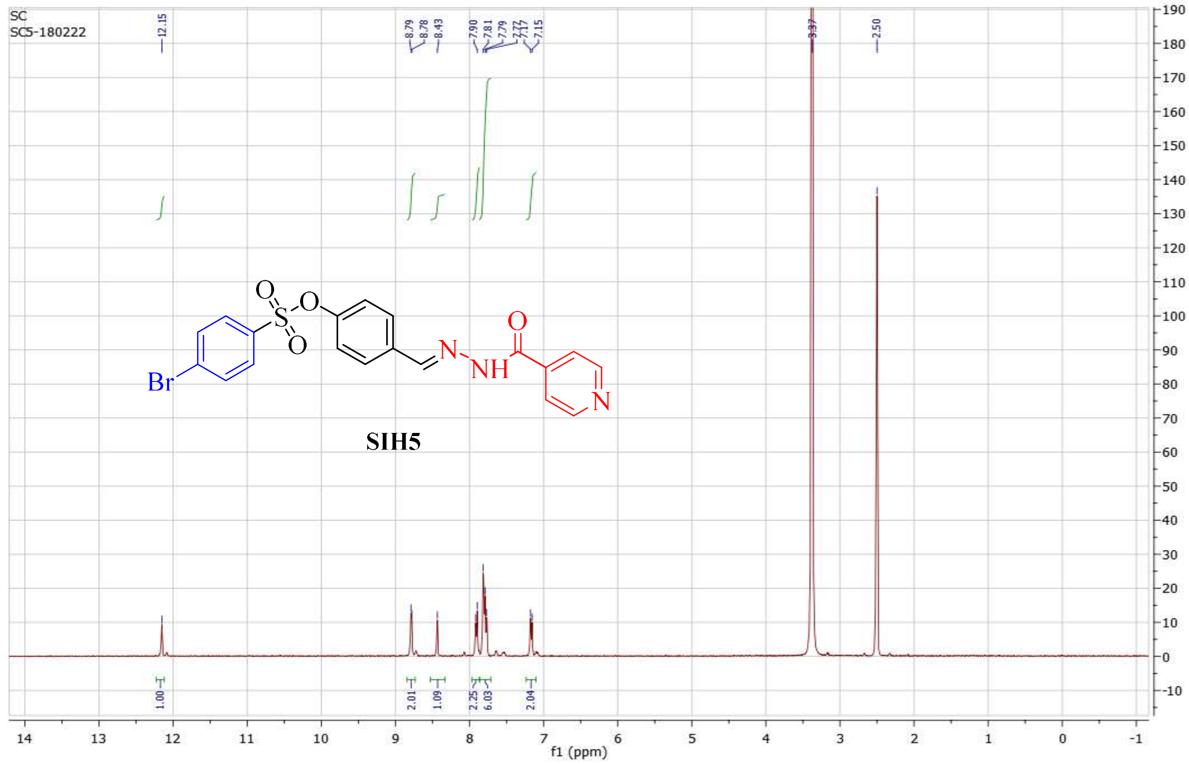

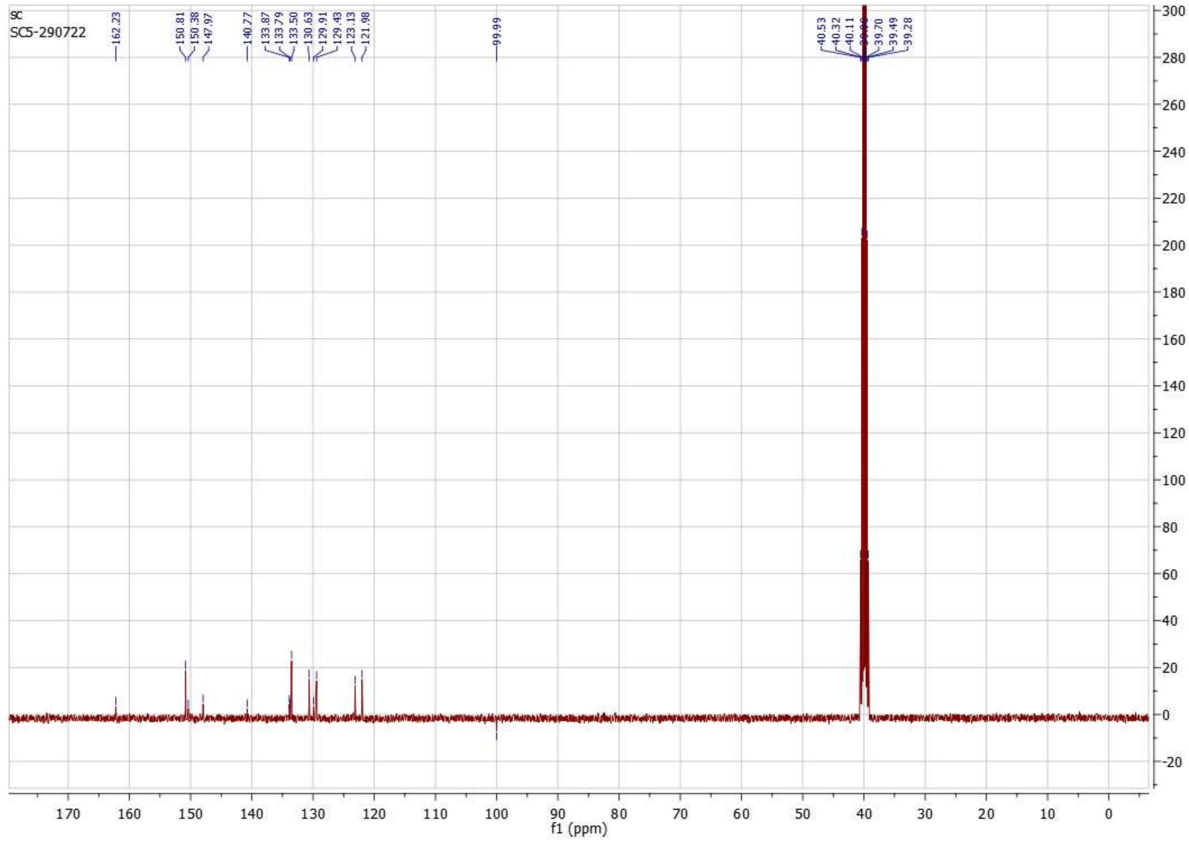

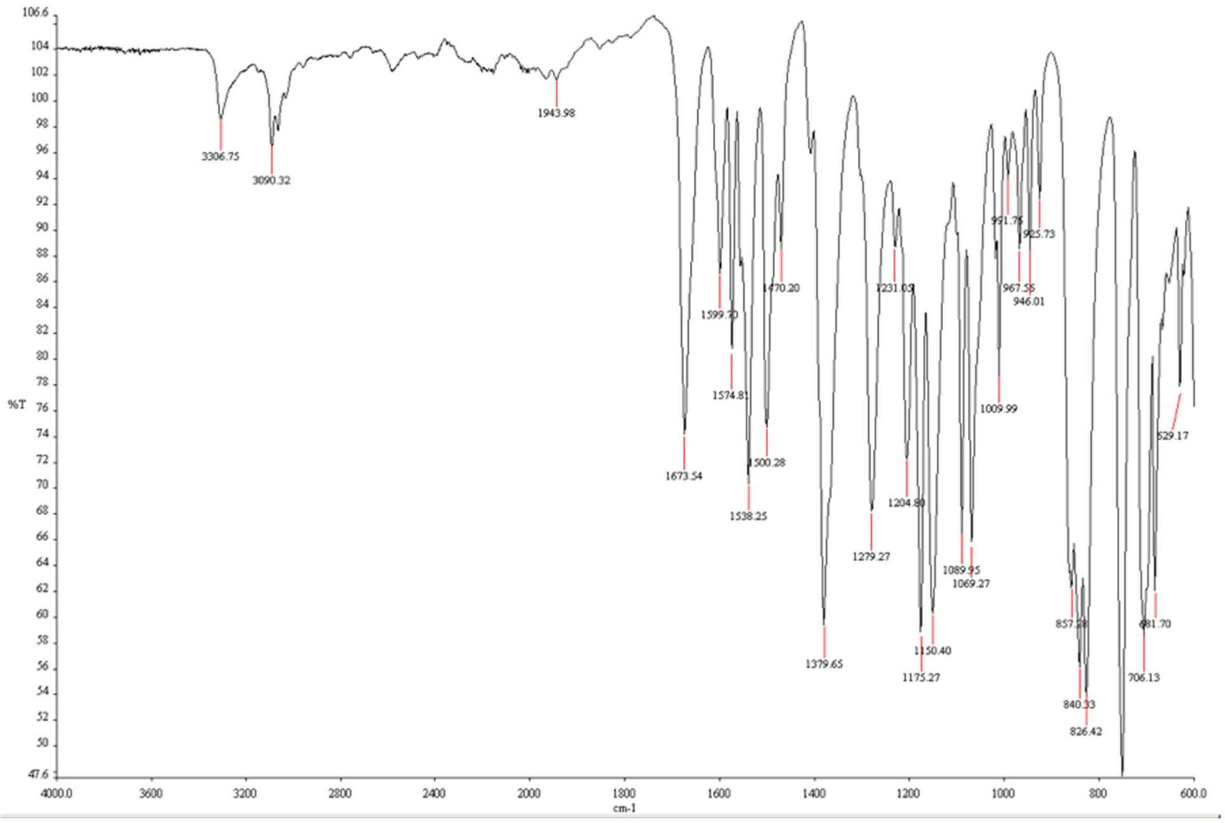

User Spectra

Fragmentor Voltage	Collision Energy	Ionization Mode
90	0	ESI

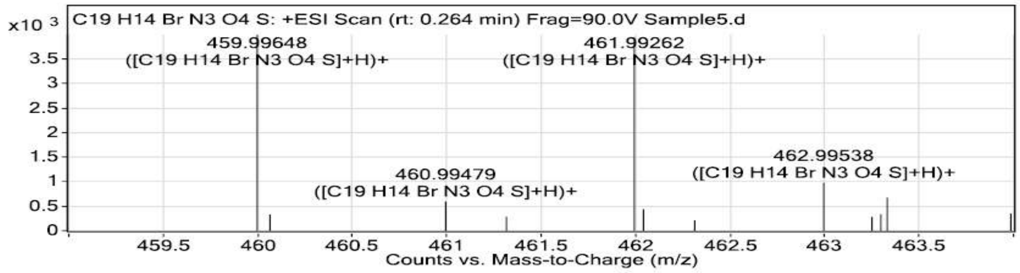

^{1}H NMR, ^{13}C NMR, HRMS and FTIR spectra of **SIH6**

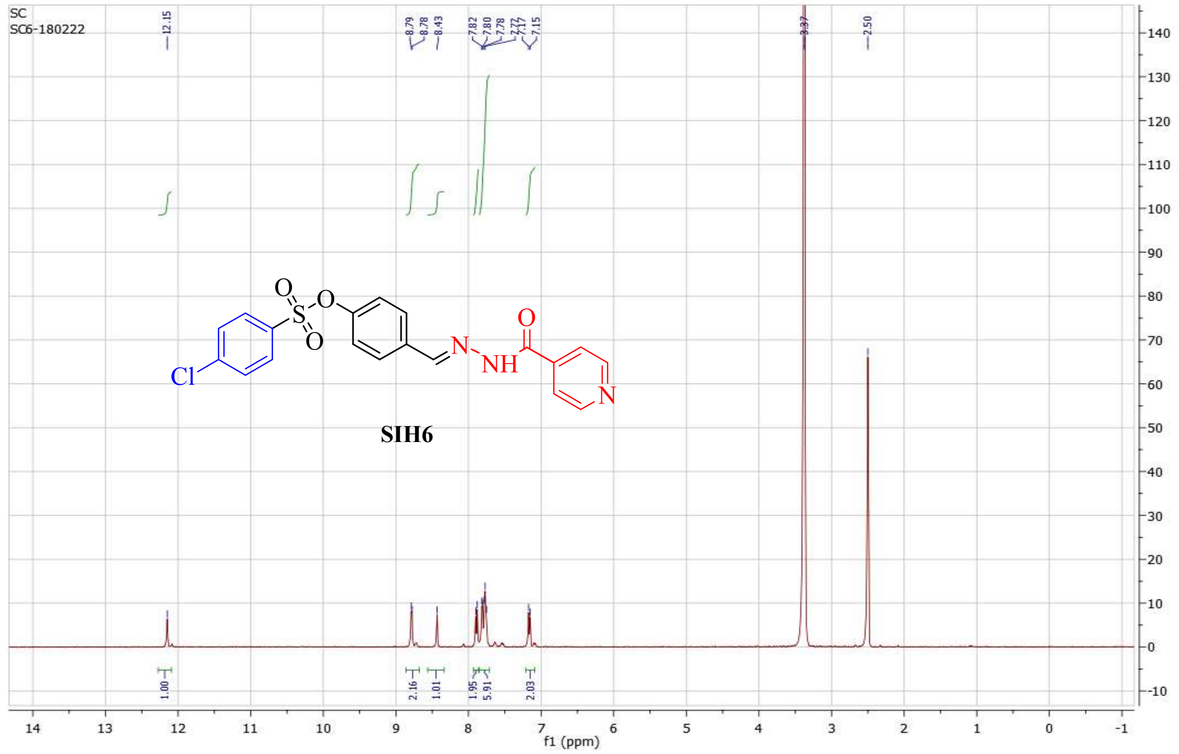

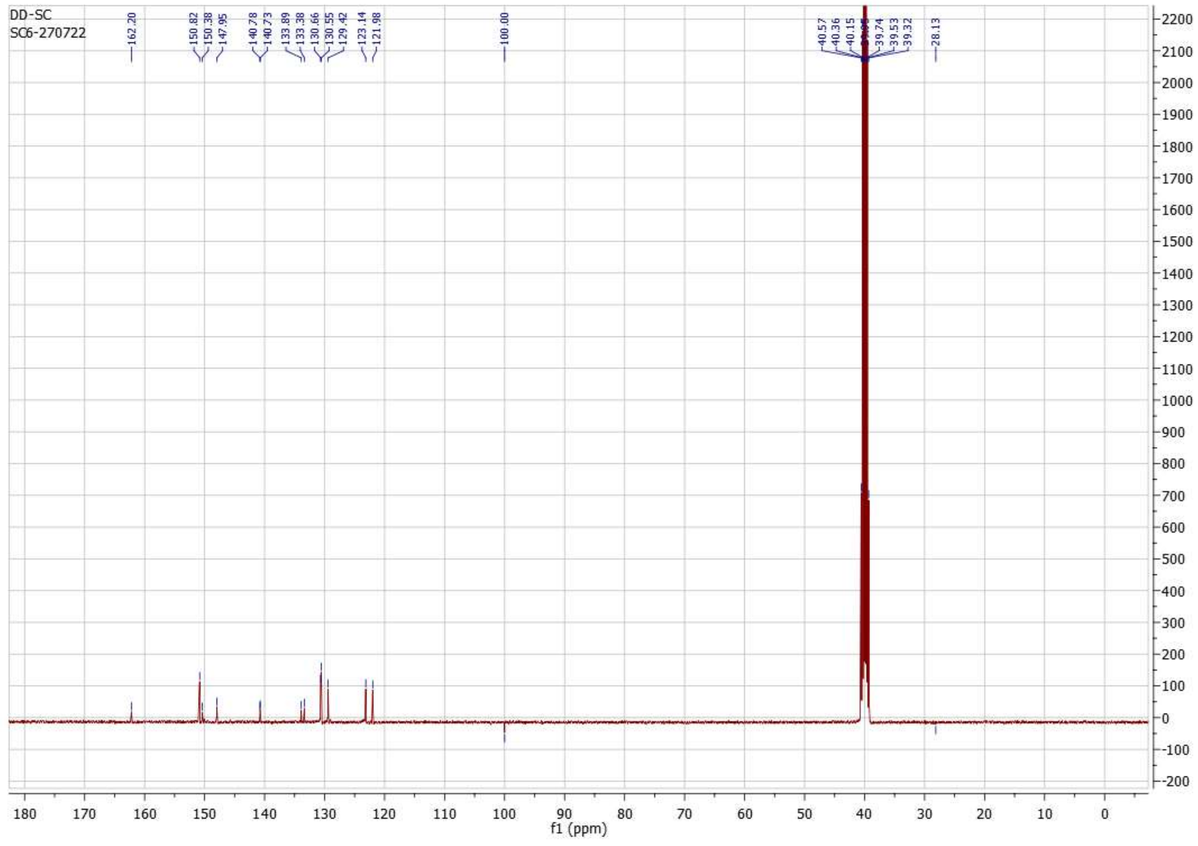

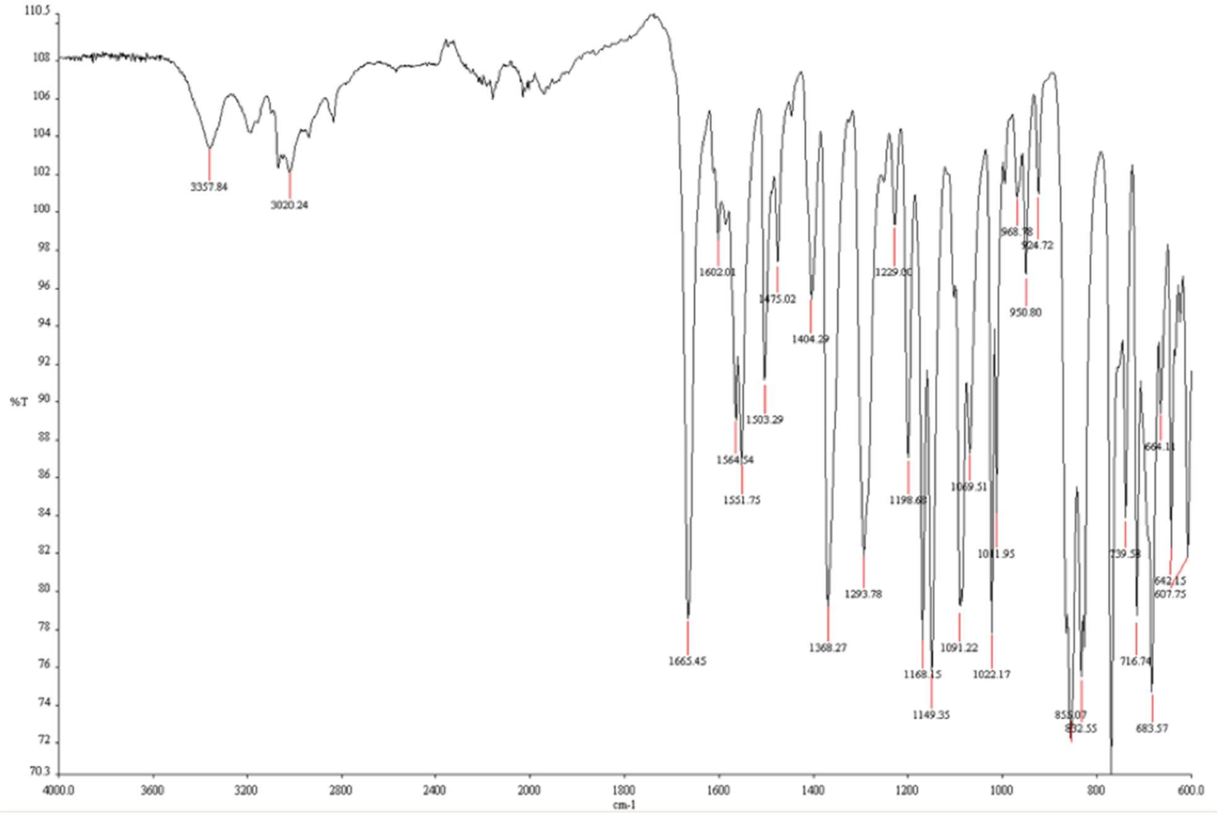
%T
cm-1
3357.84
3020.24
1665.45
1602.01
1564.54
1551.75
1503.29
1475.02
1404.29
1368.27
1293.78
1198.68
1168.15
1149.35
1091.22
1069.51
1022.17
968.78
950.80
924.72
855.07
832.55
739.58
716.74
683.57
664.11
642.15
607.75

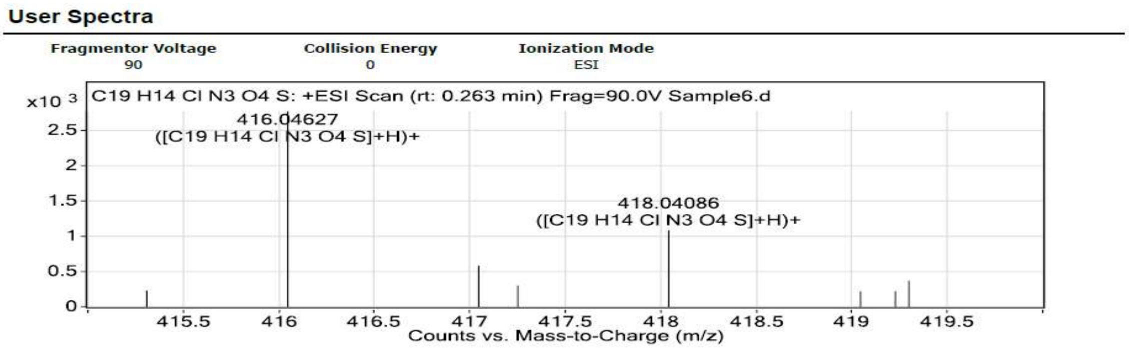
User Spectra
Fragmentor Voltage
90
Collision Energy
0
Ionization Mode
ESI
C19 H14 Cl N3 O4 S: +ESI Scan (rt: 0.263 min) Frag=90.0V Sample6.d
x10 3
416.04627
([C19 H14 Cl N3 O4 S]+H)+
418.04086
([C19 H14 Cl N3 O4 S]+H)+
Counts vs. Mass-to-Charge (m/z)

^{1}H NMR, ^{13}C NMR, HRMS and FTIR spectra of **SIH7**

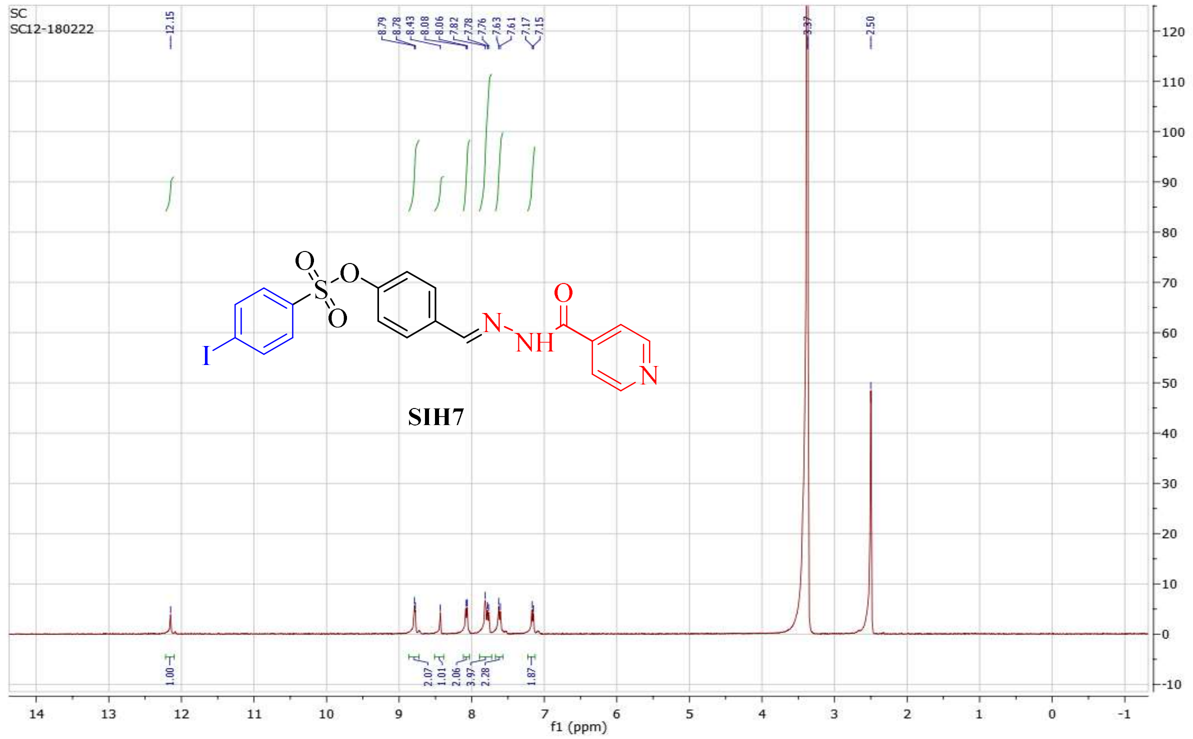

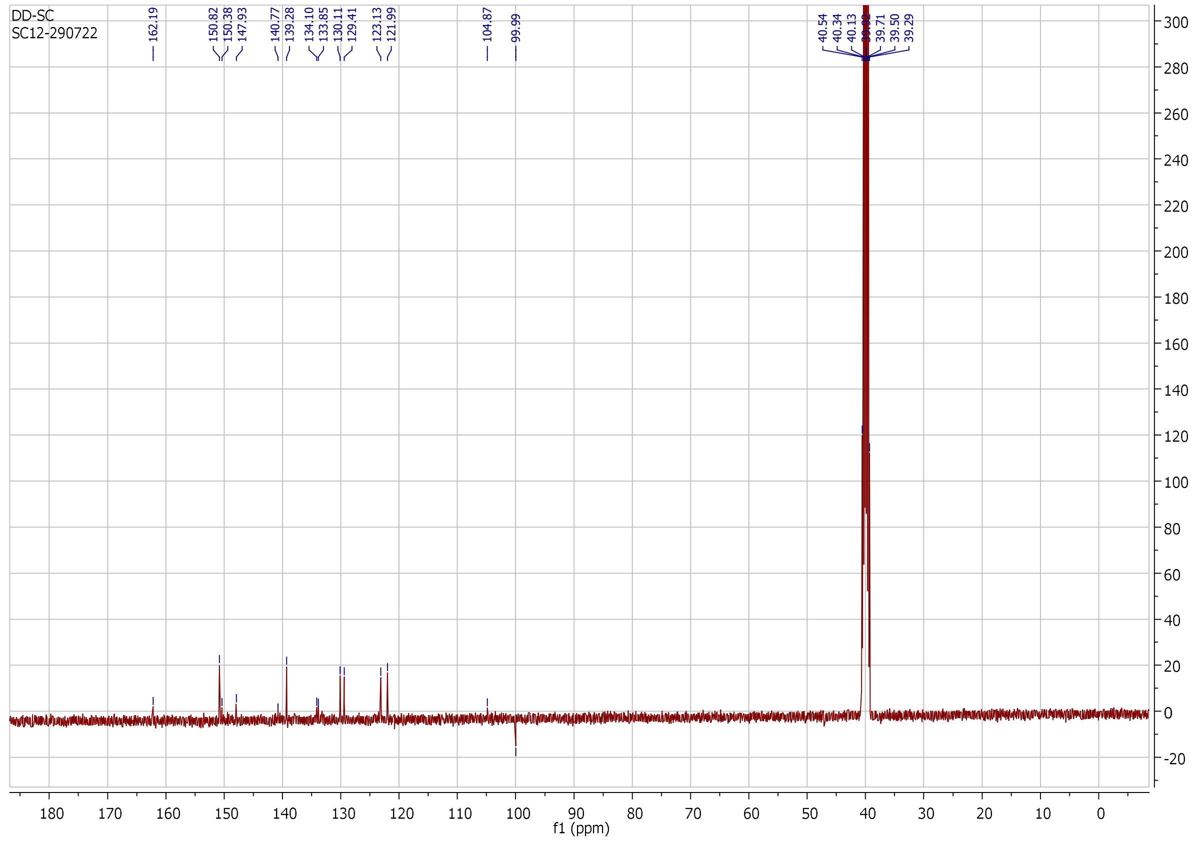

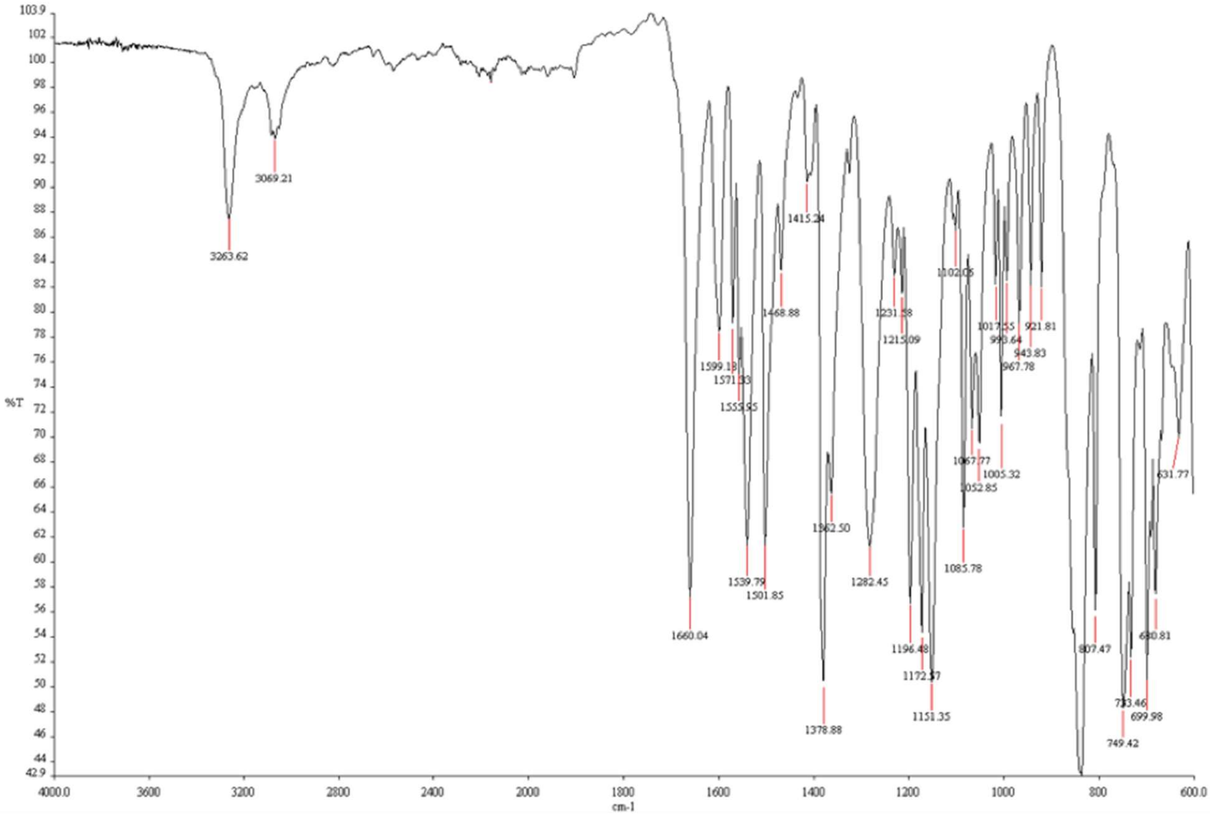
%T
cm-1
3263.62
3069.21
1660.04
1599.13
1539.79
1501.85
1468.88
1415.24
1378.88
1362.50
1282.45
1231.58
1215.09
1196.48
1172.57
1151.35
1102.05
1085.78
1067.77
1052.85
1017.55
1005.32
967.78
943.83
921.81
807.47
749.42
699.98
680.81
631.77

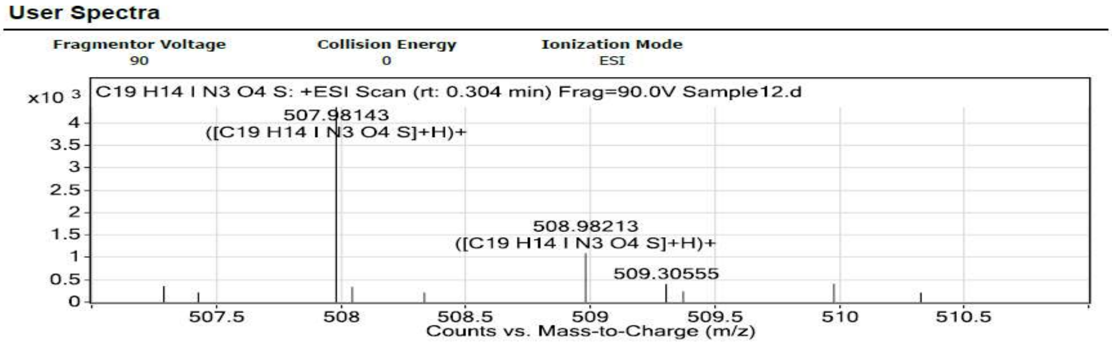
User Spectra
Fragmentor Voltage
90
Collision Energy
0
Ionization Mode
ESI
C19 H14 I N3 O4 S: +ESI Scan (rt: 0.304 min) Frag=90.0V Sample12.d
x10 3
507.98143
([C19 H14 I N3 O4 S]+H)+
508.98213
([C19 H14 I N3 O4 S]+H)+
509.30555
Counts vs. Mass-to-Charge (m/z)

^{1}H NMR, ^{13}C NMR, HRMS and FTIR spectra of **SIH8**

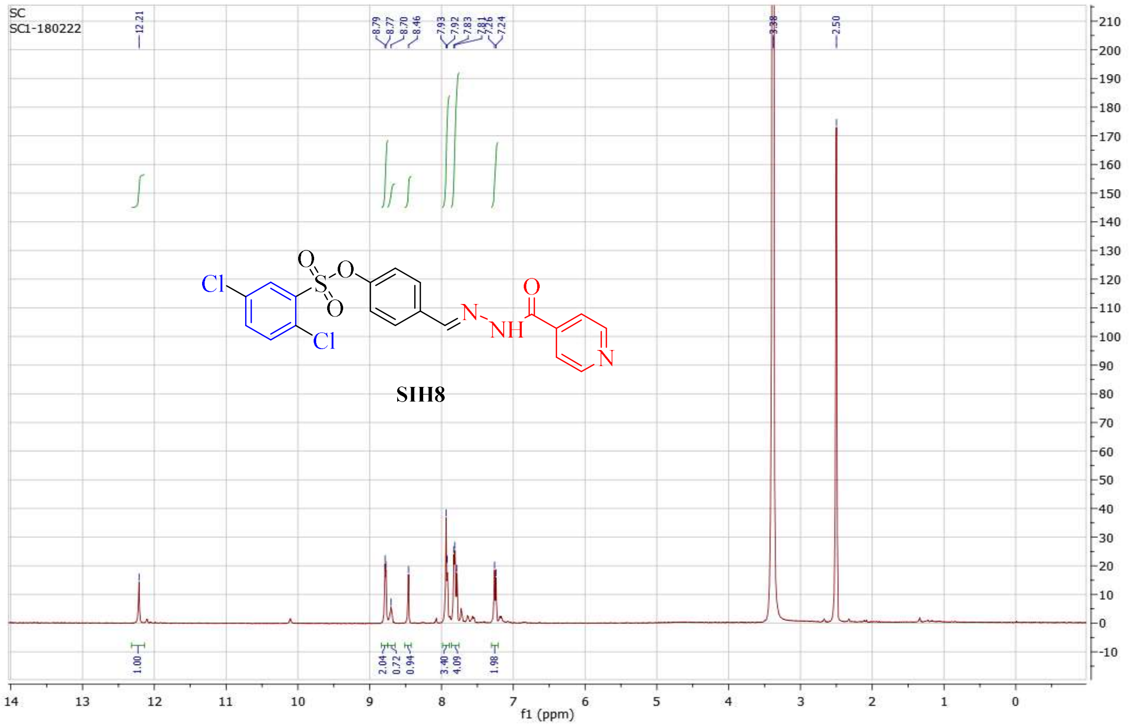

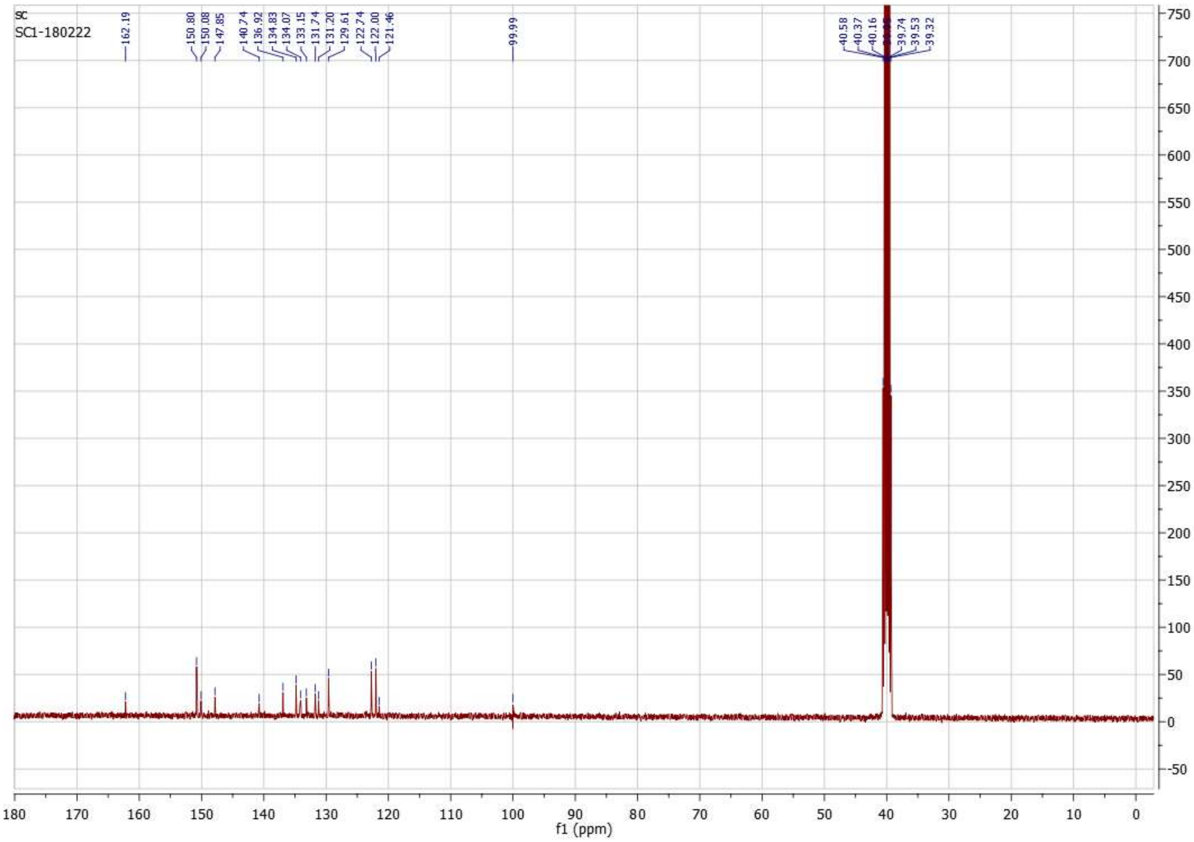

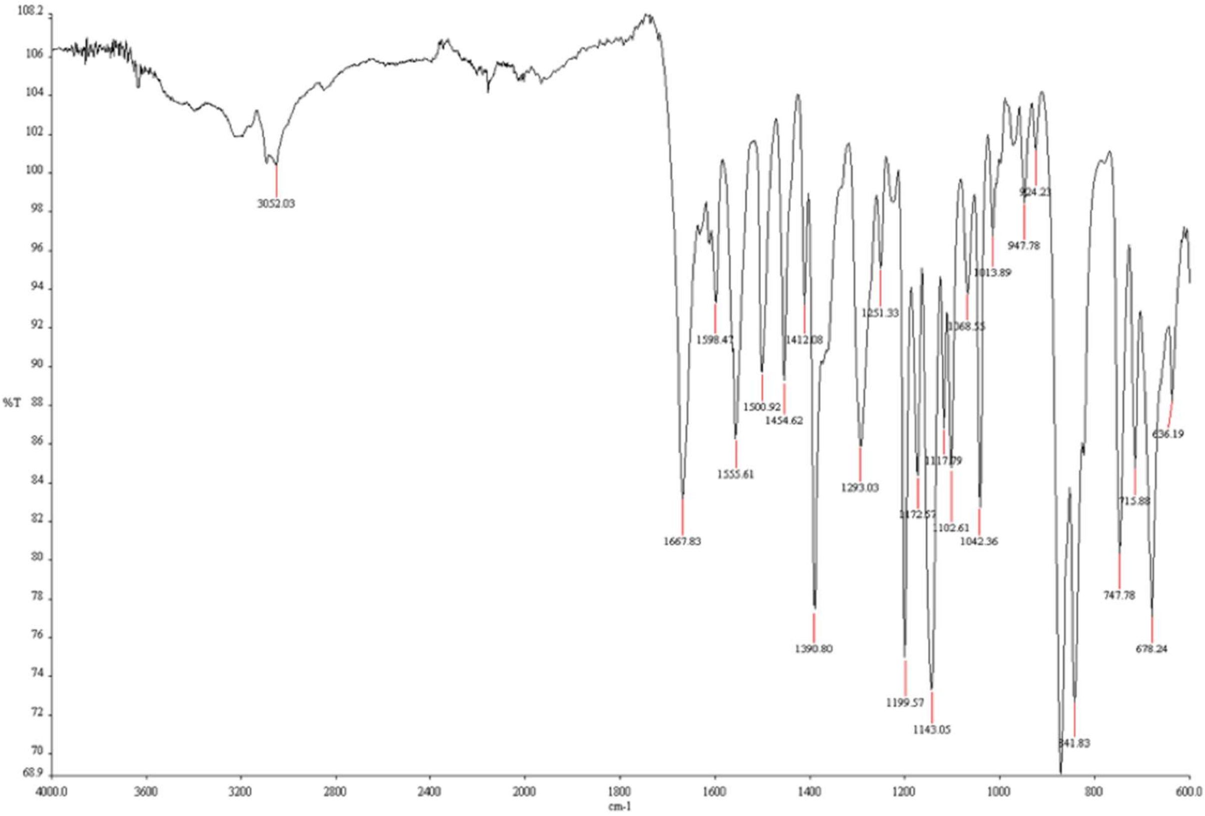
%T
cm-1
3052.03
1667.83
1598.47
1555.61
1500.92
1454.62
1412.08
1390.80
1293.03
1251.33
1199.57
1172.57
1143.05
1117.79
1102.61
1068.55
1042.36
1013.89
947.78
924.23
841.83
747.78
715.88
678.24
636.19

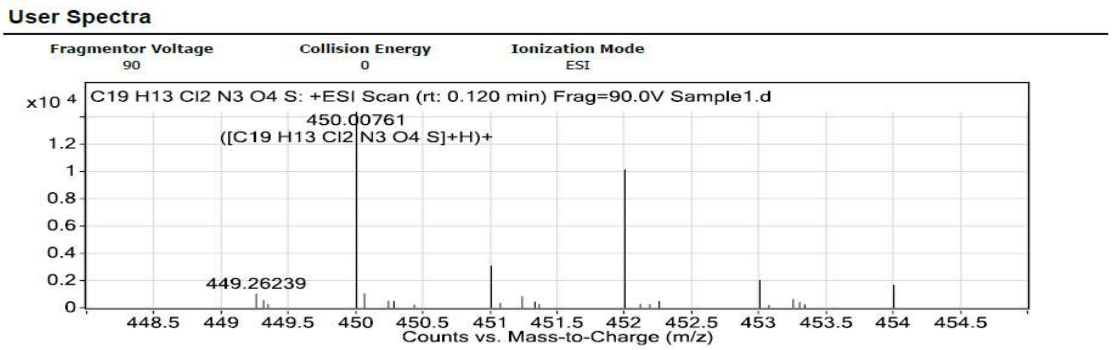
User Spectra
Fragmentor Voltage
90
Collision Energy
0
Ionization Mode
ESI
C19 H13 Cl2 N3 O4 S: +ESI Scan (rt: 0.120 min) Frag=90.0V Sample1.d
450.00761
([C19 H13 Cl2 N3 O4 S]+H)+
449.26239
Counts vs. Mass-to-Charge (m/z)

^{1}H NMR, ^{13}C NMR, HRMS and FTIR spectra of **SIH9**

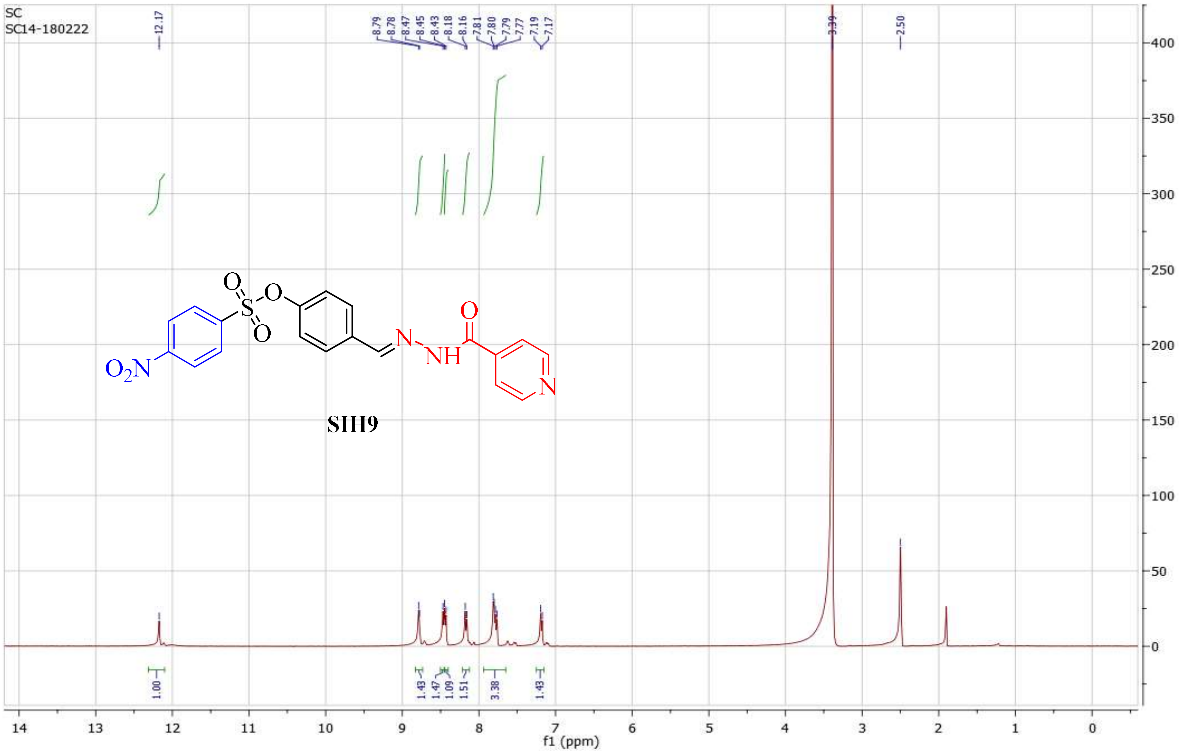

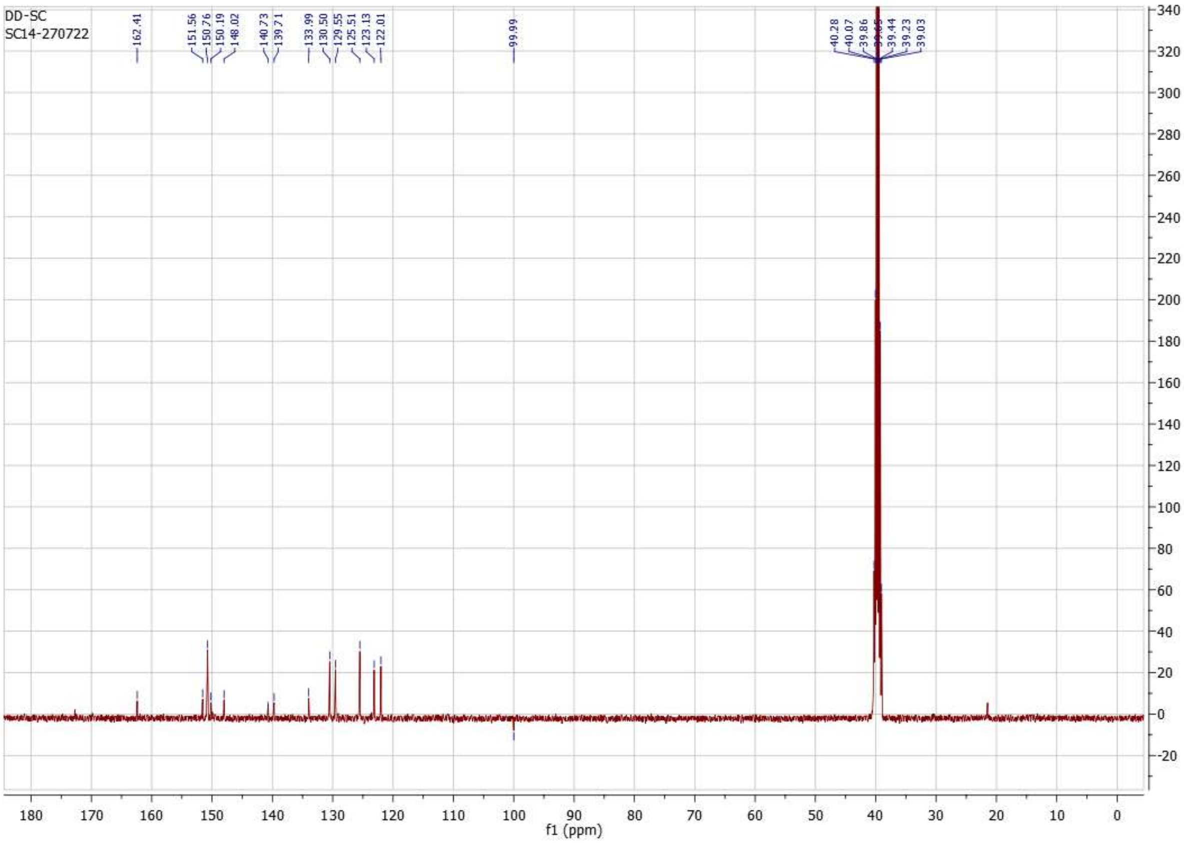

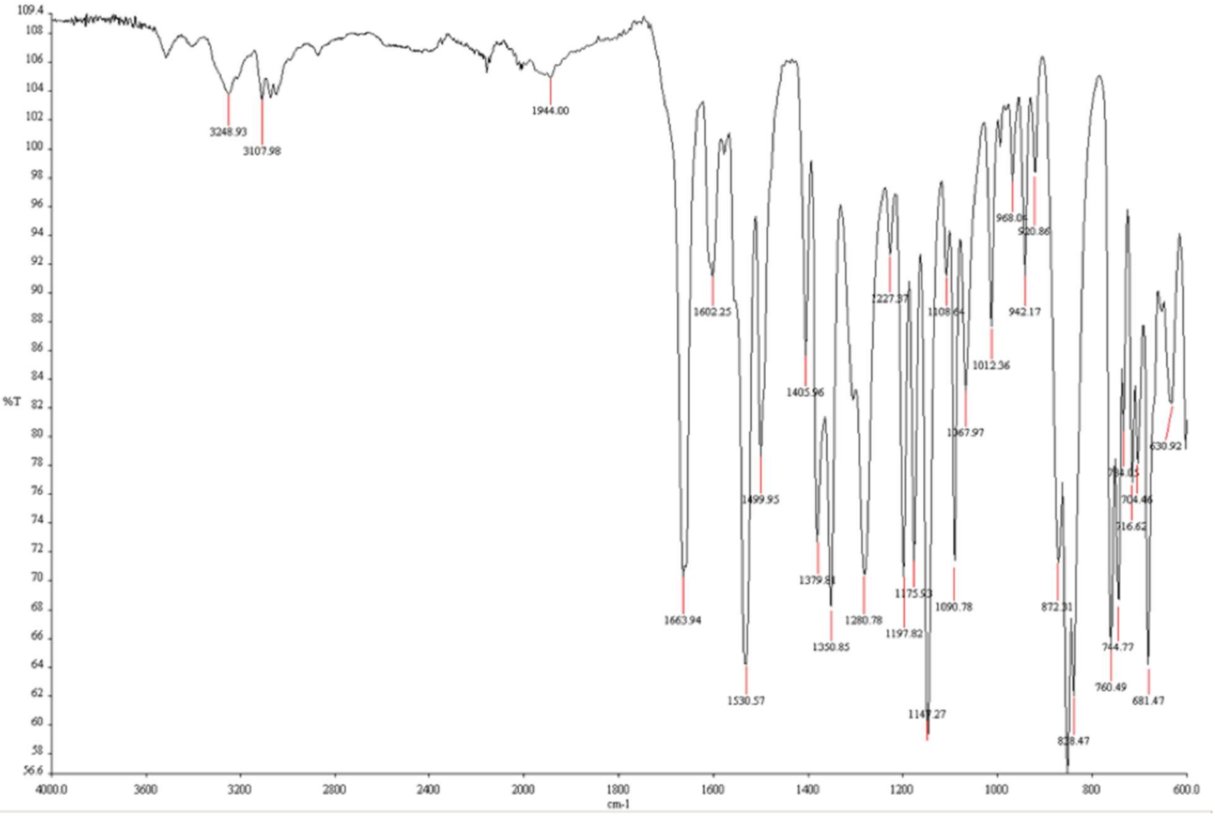

User Spectra

Fragmentor Voltage	Collision Energy	Ionization Mode
90	0	ESI

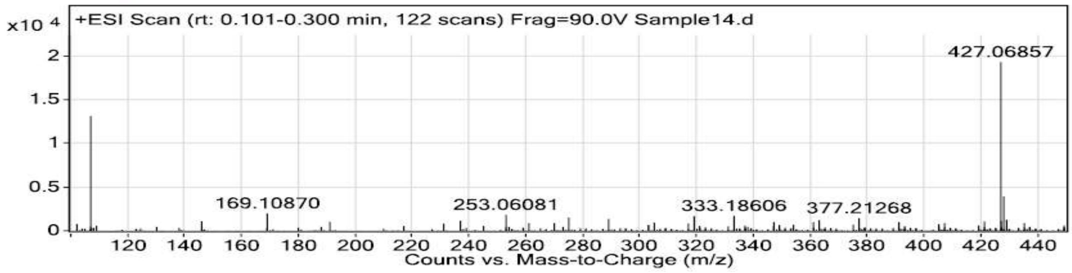

^{1}H NMR, ^{13}C NMR, HRMS and FTIR spectra of **SIH10**

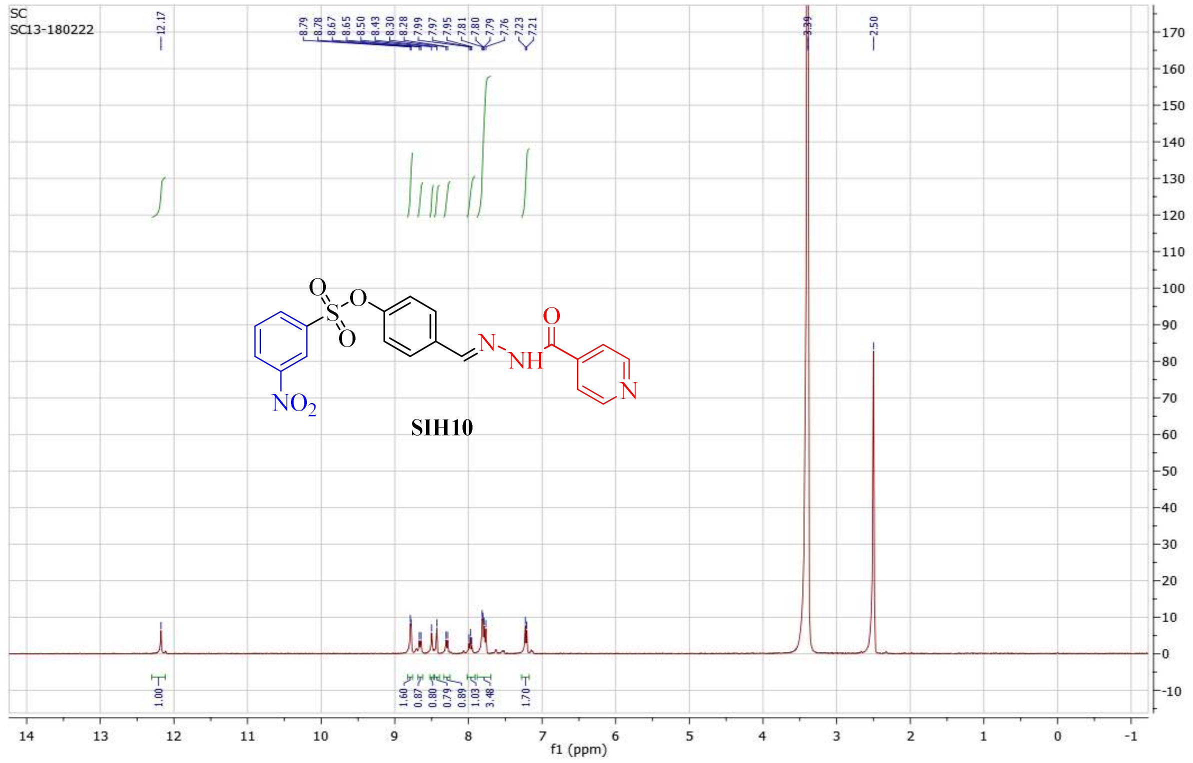

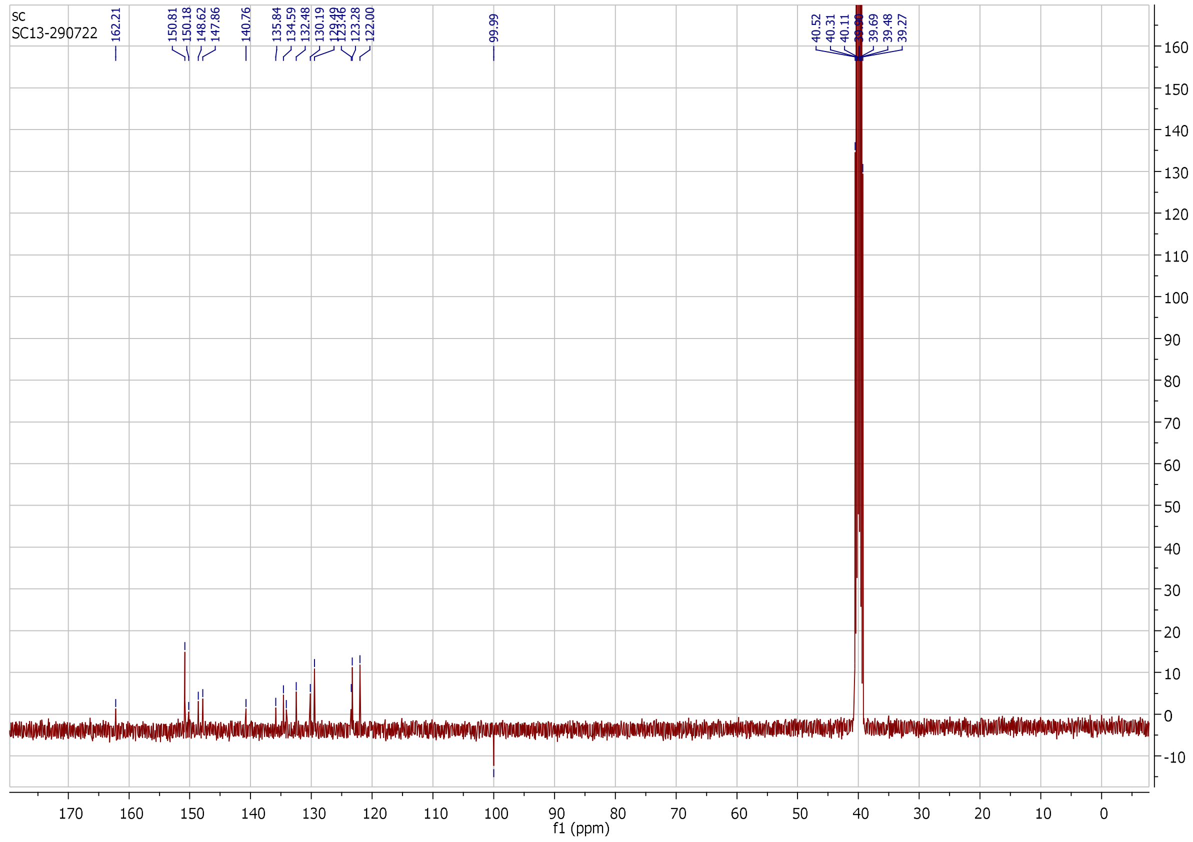

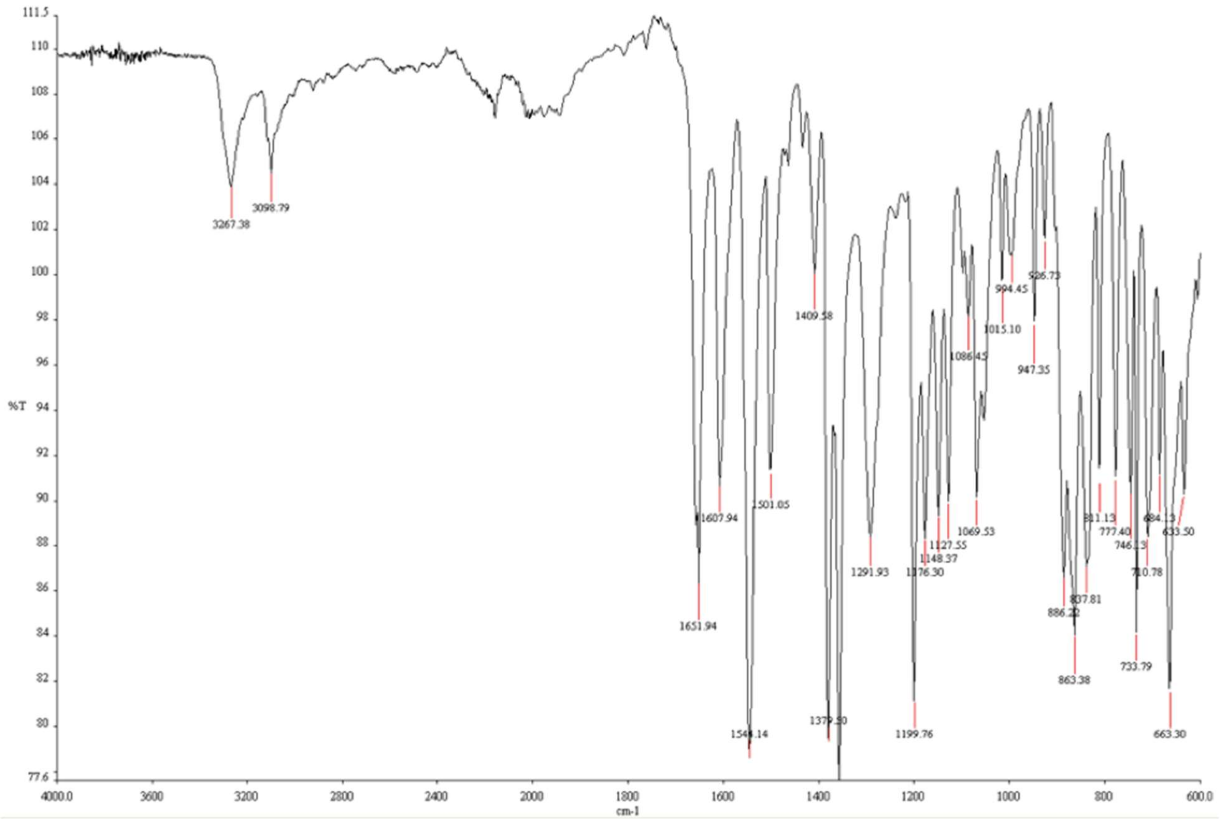

User Spectra

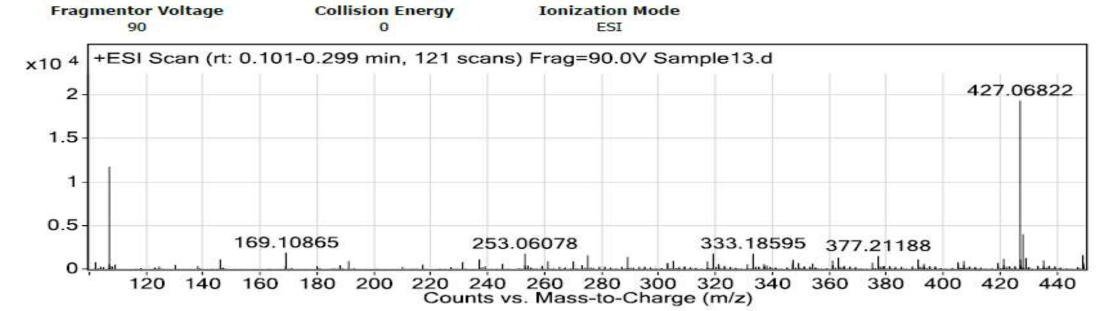

^{1}H NMR, ^{13}C NMR, HRMS and FTIR spectra of **SIH11**

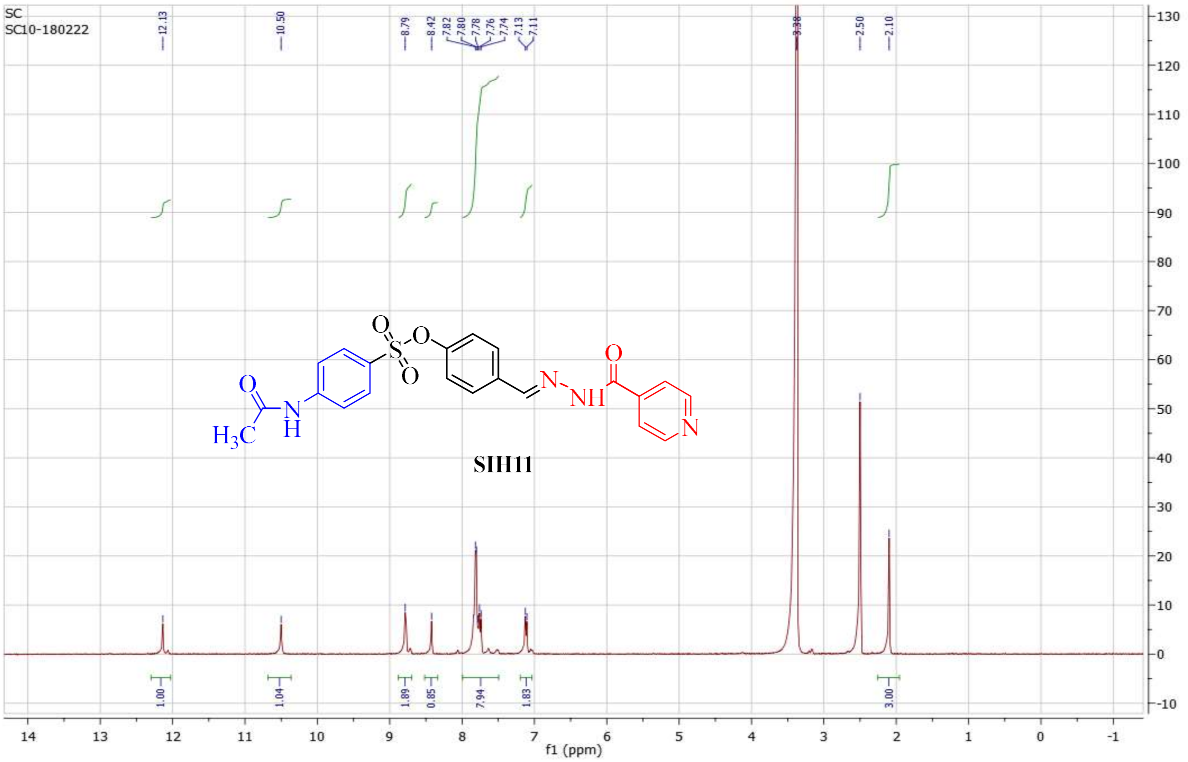

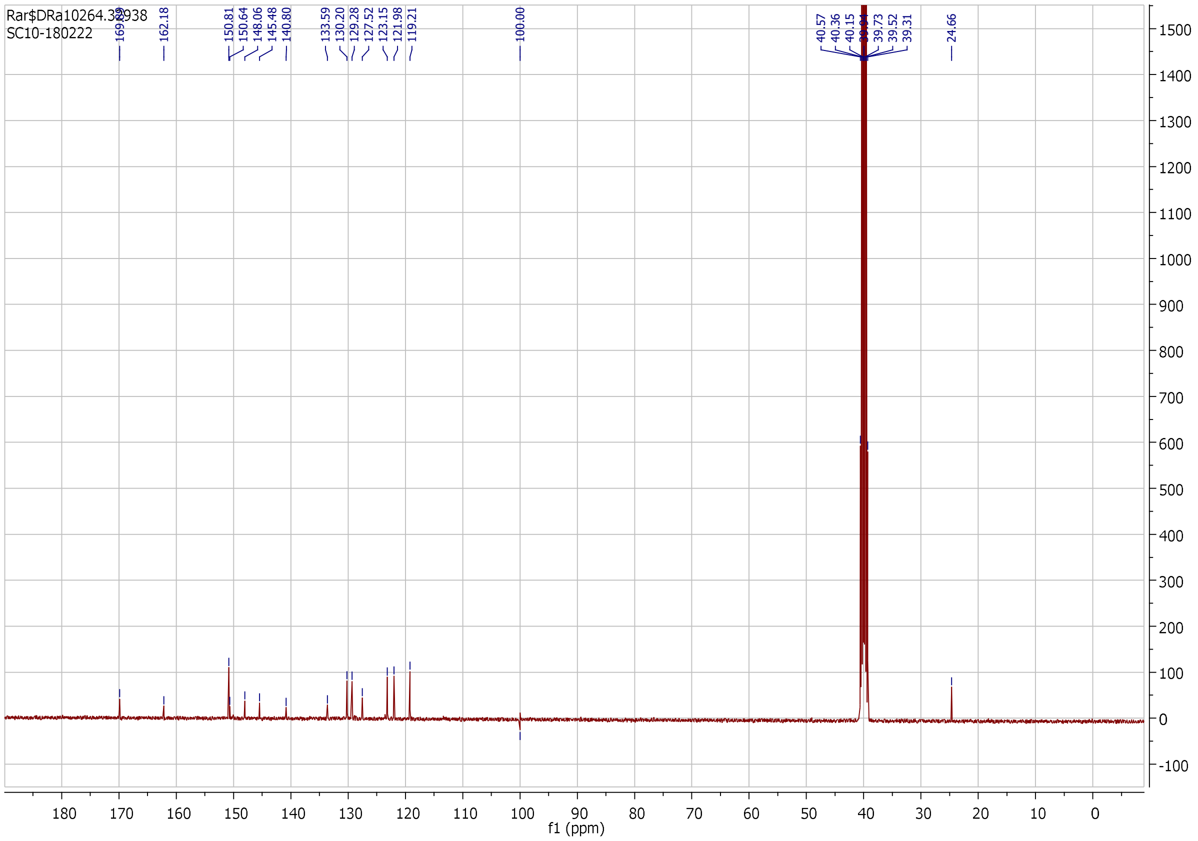

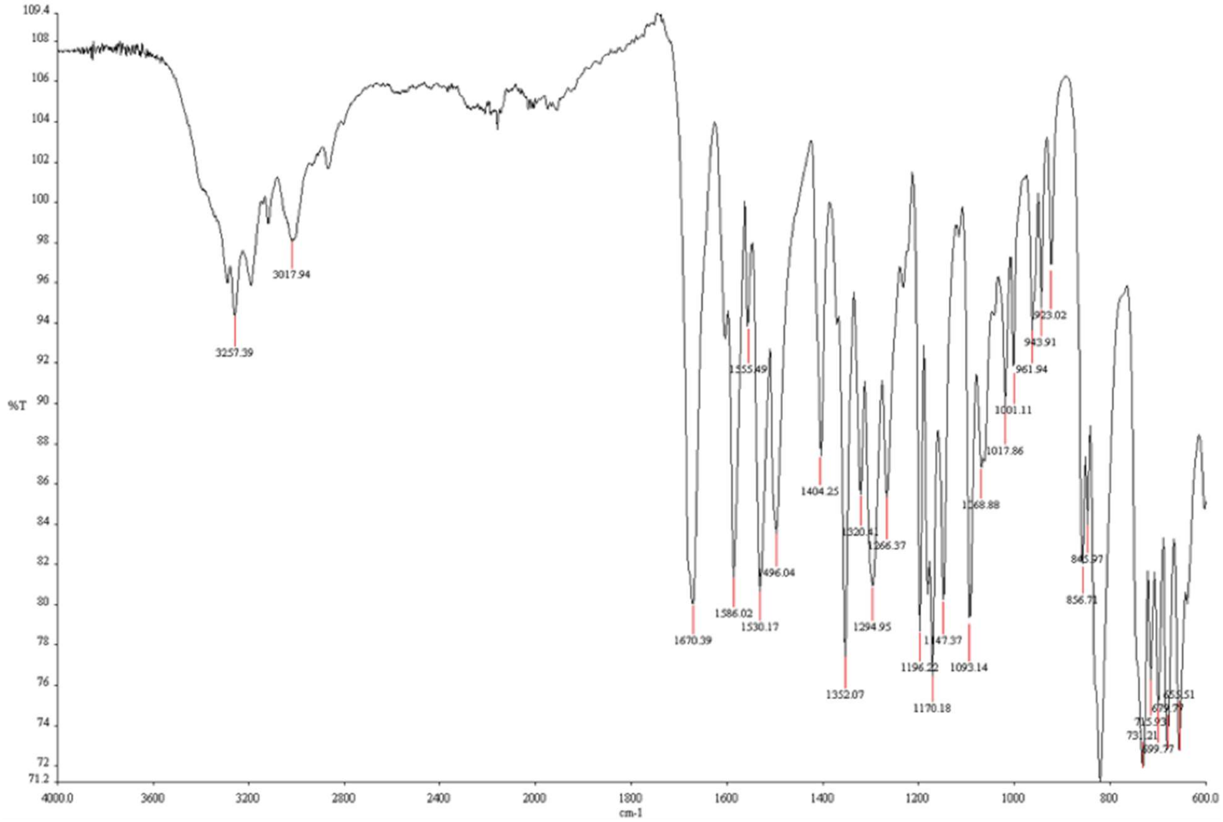
%T
cm-1
3257.39
3017.94
1670.39
1586.02
1555.49
1530.17
1496.04
1404.25
1352.07
1294.95
1266.37
1196.22
1170.18
1147.37
1093.14
1017.86
1001.11
961.94
943.91
923.02
856.71

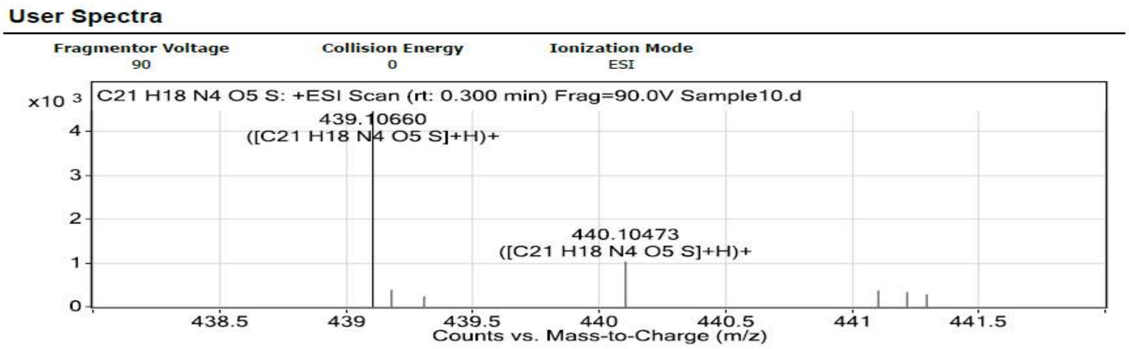
User Spectra
Fragmentor Voltage
90
Collision Energy
0
Ionization Mode
ESI
C21 H18 N4 O5 S: +ESI Scan (rt: 0.300 min) Frag=90.0V Sample10.d
x10 3
439.10660
([C21 H18 N4 O5 S]+H)+
440.10473
([C21 H18 N4 O5 S]+H)+
Counts vs. Mass-to-Charge (m/z)

^{1}H NMR, ^{13}C NMR, HRMS and FTIR spectra of **SIH12**

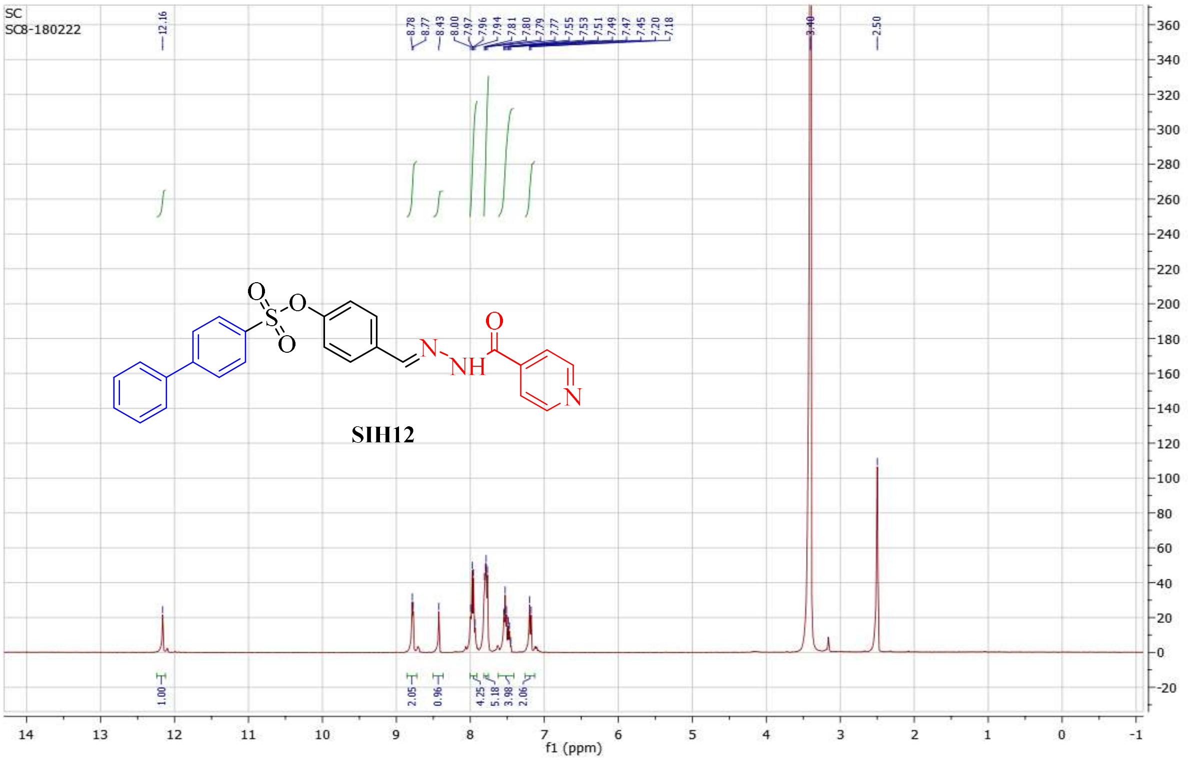

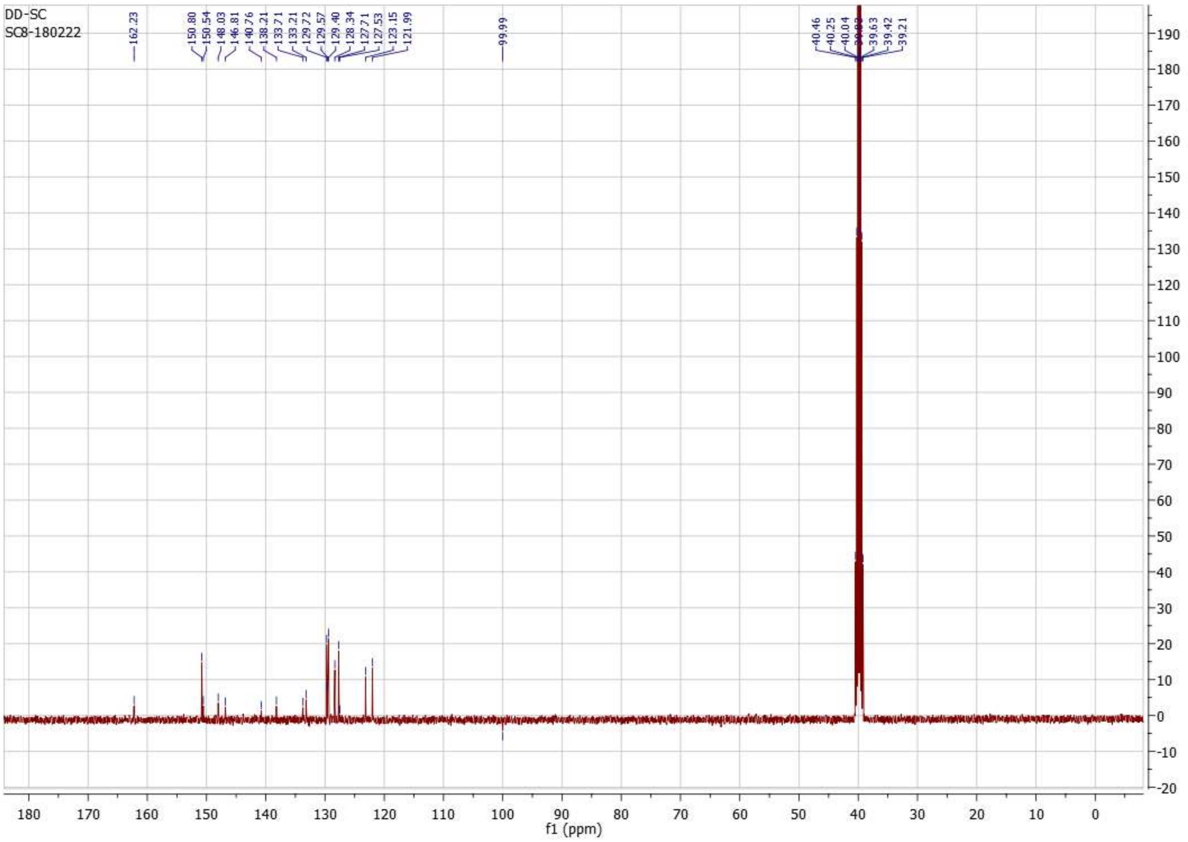

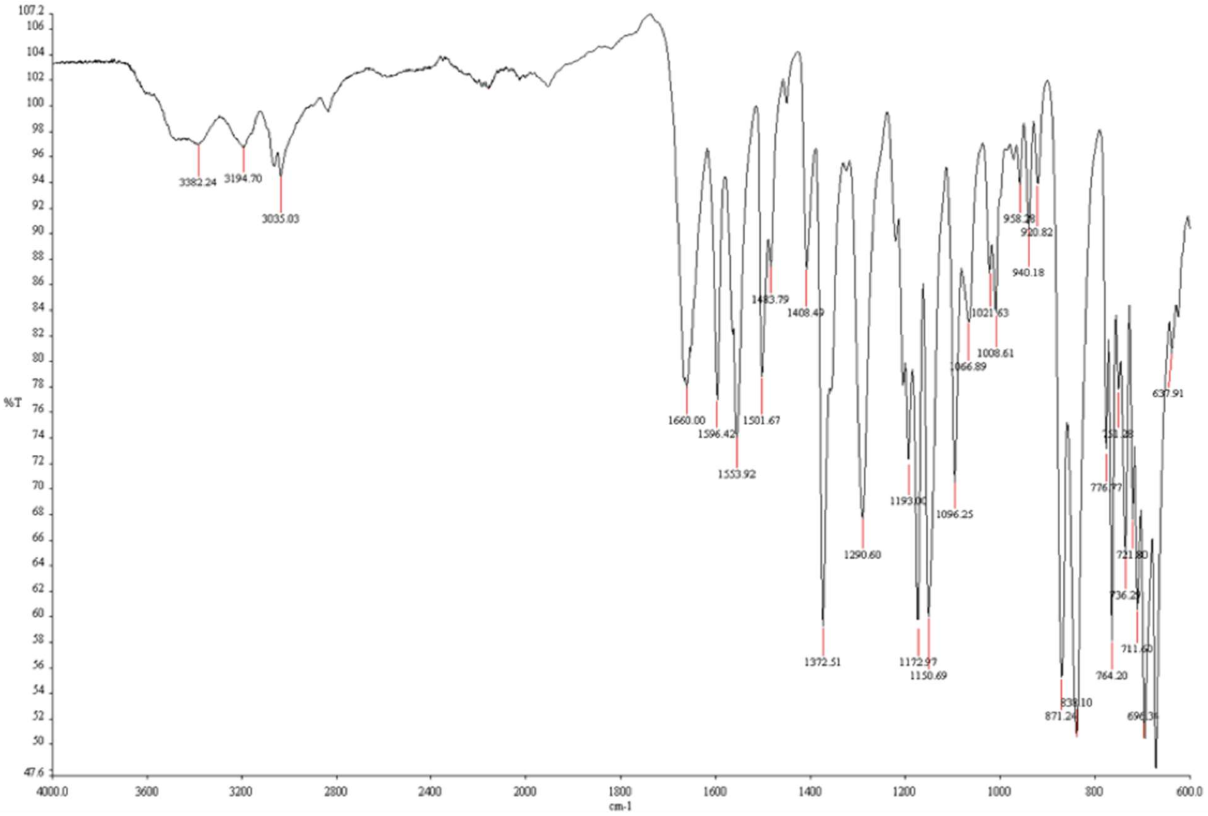

%T
cm-1
3382.24
3194.70
3035.03
1660.00
1596.42
1553.92
1501.67
1483.79
1408.49
1372.51
1290.60
1193.00
1172.97
1150.69
1096.25
1066.89
1021.63
1008.61
958.28
940.18
920.82
871.24
838.10
776.77
764.20
736.29
721.80
711.60
696.38
637.91

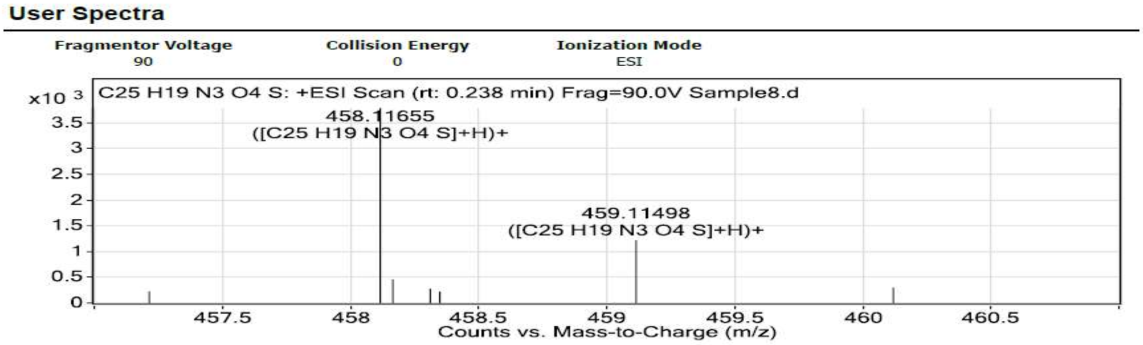

User Spectra
Fragmentor Voltage 90
Collision Energy 0
Ionization Mode ESI
C25 H19 N3 O4 S: +ESI Scan (rt: 0.238 min) Frag=90.0V Sample8.d
x10 3
458.11655
([C25 H19 N3 O4 S]+H)+
459.11498
([C25 H19 N3 O4 S]+H)+
Counts vs. Mass-to-Charge (m/z)

^{1}H NMR, ^{13}C NMR, HRMS and FTIR spectra of **SIH13**

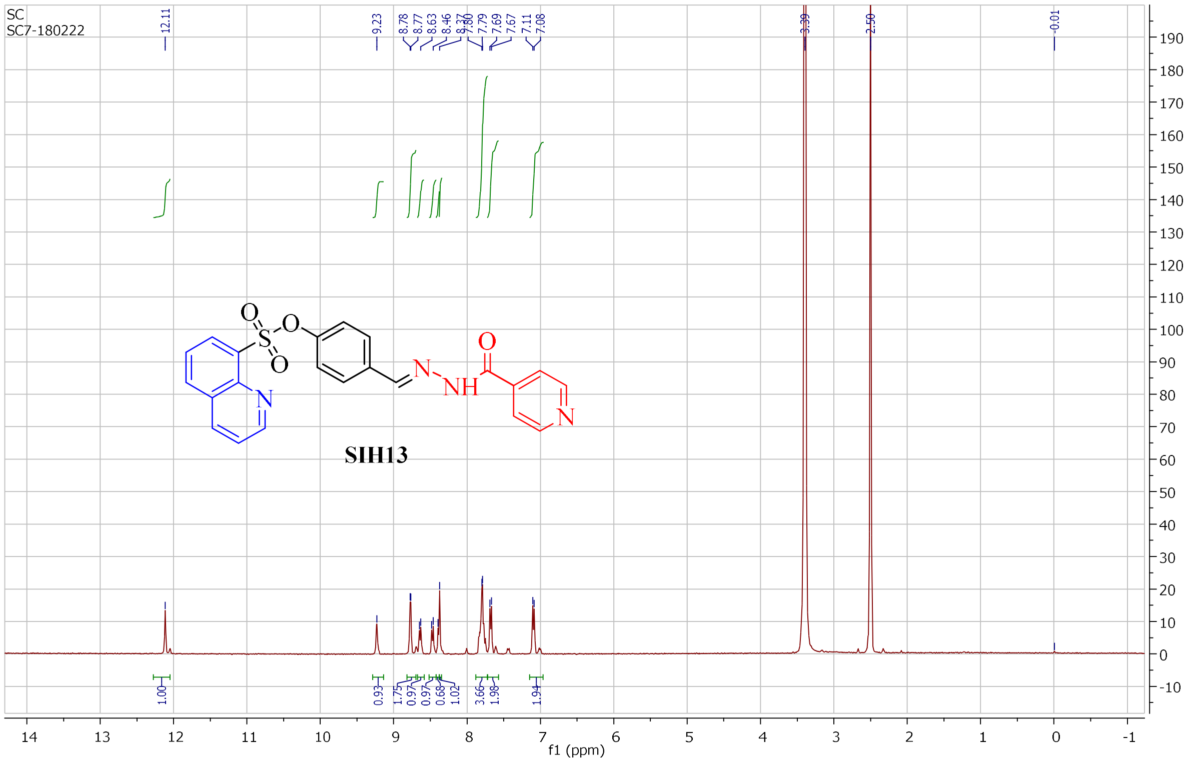

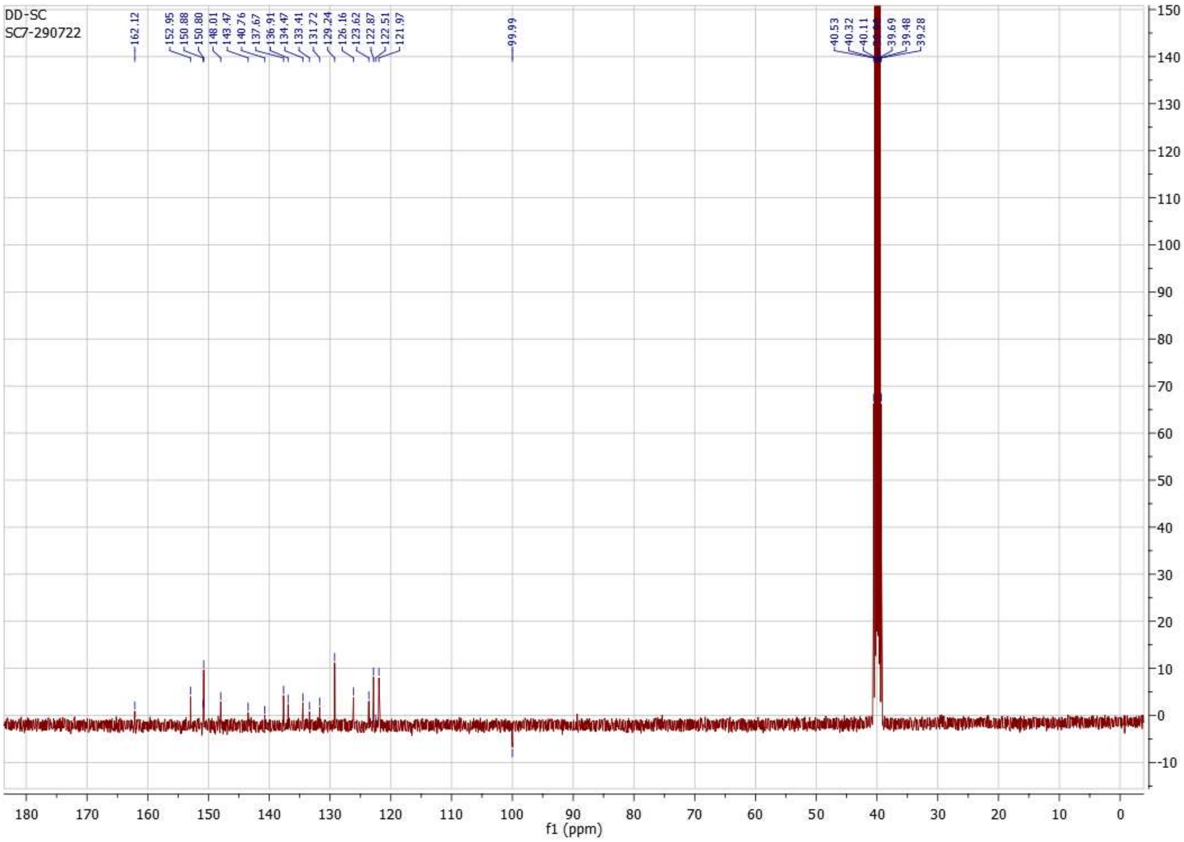

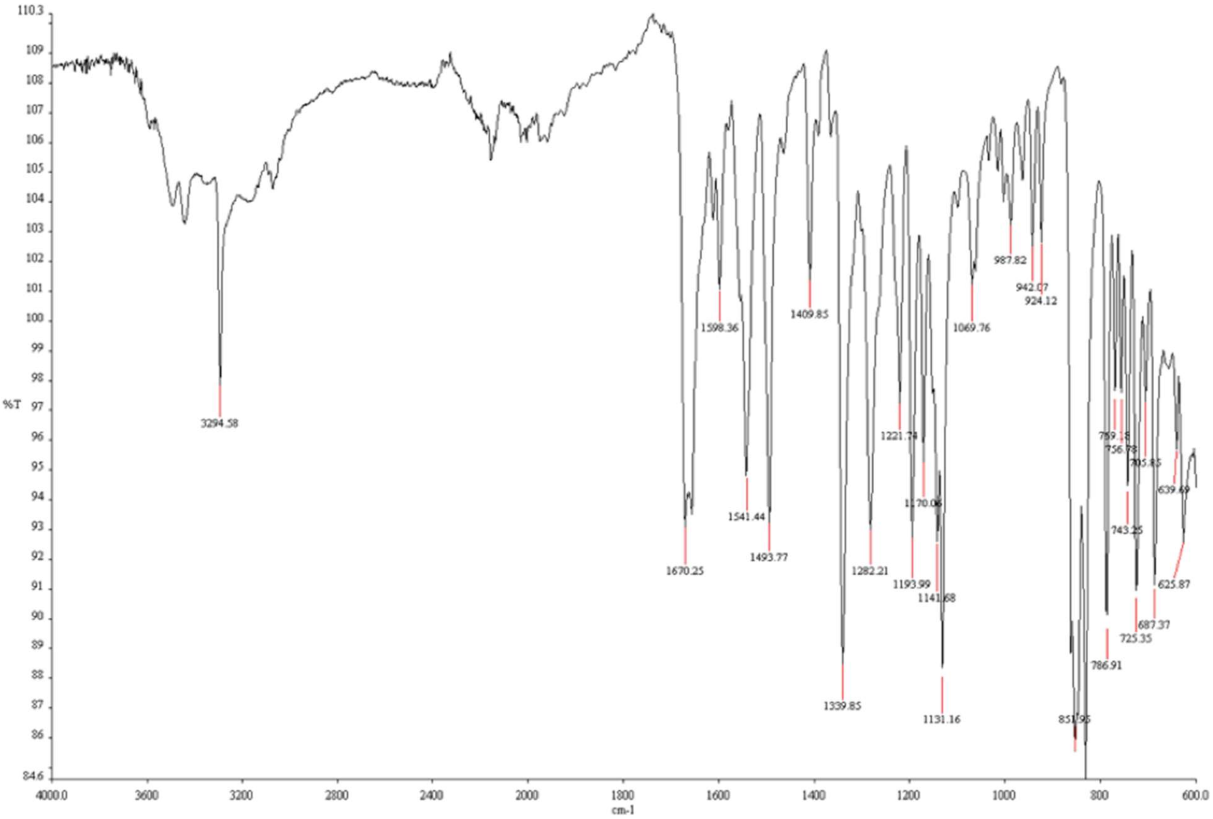
%T
cm-1
3294.58
1670.25
1598.36
1541.44
1493.77
1409.85
1339.85
1282.21
1221.74
1193.99
1170.06
1141.68
1131.16
1069.76
987.82
942.07
924.12
786.91
743.25
725.35
705.85
687.37
639.69
625.87

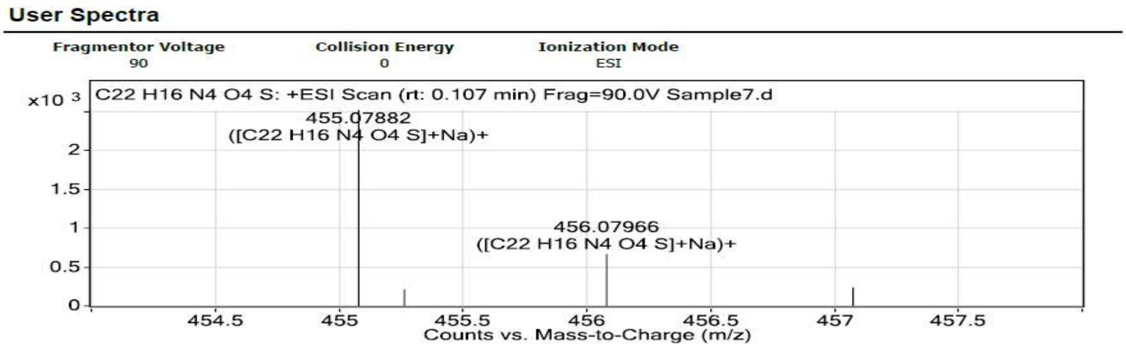
User Spectra
Fragmentor Voltage
90
Collision Energy
0
Ionization Mode
ESI
C22 H16 N4 O4 S: +ESI Scan (rt: 0.107 min) Frag=90.0V Sample7.d
x10 3
455.07882
([C22 H16 N4 O4 S]+Na)+
456.07966
([C22 H16 N4 O4 S]+Na)+
Counts vs. Mass-to-Charge (m/z)

InhA optimised sequence:

ATGACAGGGCTATTAGATGGAAAAAGGATATTGGTCTCCGGTATCATCACTGACT
CTTCTATCGCATTTCACATCGCGCGCGTTGCCCAAGAGCAGGGTGCGCAACTGGT
GCTGACCGGCTTCGACCGCCTAAGACTTATCCAACGTATTACCGACCGTCTGCCG
GCTAAGGCTCCGCTGCTTGAGCTGGACGTTCAGAATGAAGAGCACCTGGCCTCCC
TGGCTGGCCGTGTGACCGAAGCGATCGGCGCGGGAAACAAACTGGACGGTGTCG
TGCACTCCATCGGCTTCATGCCGCAGACCGGTATGGGTATTAACCCGTTTTTCGA
CGCACCGTATGCCGACGTGTCGAAAGGTATTCATATTAGCGCATACAGCTATGCG
AGCATGGCGAAGGCGCTCTTGCCTATCATGAACCCGGGTGGCTCGATCGTGGGCA
TGGATTTTGATCCGAGCCGTGCAATGCCGGCGTACAACTGGATGACCGTTGCGAA
GTCTGCGTTGGAGAGCGTTAATCGTTTCGTGGCCCGTGAAGCTGGCAAATACGGC
GTTCGTAGCAATCTGGTAGCGGCAGGTCCGATCCGCACCCTGGCGATGAGCGCG
ATTGTTGGTGGCGCGTTAGGTGAAGAGGCTGGCGCTCAAATTCAGCTGCTGGAAG
AGGGTTGGGATCAGCGCGCGCCAATTGGTTGGAACATGAAAGATGCCACTCCGG
TTGCTAAGACGGTGTGCGCATTGCTGAGCGATTGGCTGCCGGCGACCACGGGTGA
TATTATCTATGCGGATGGTGGCGCGCATACCCAGCTGTTG

Stability studies in water and DMSO

a)

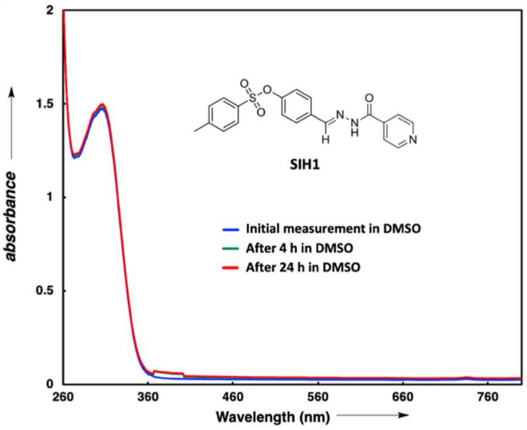

b)

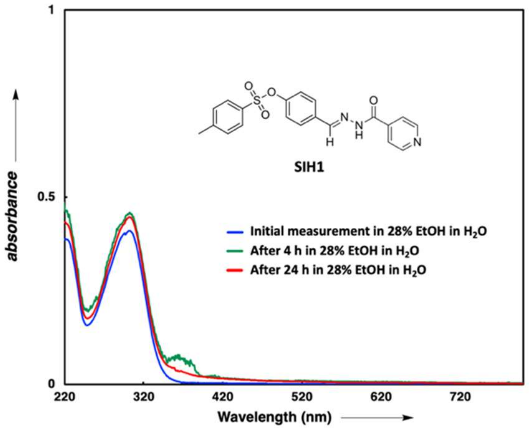

a)

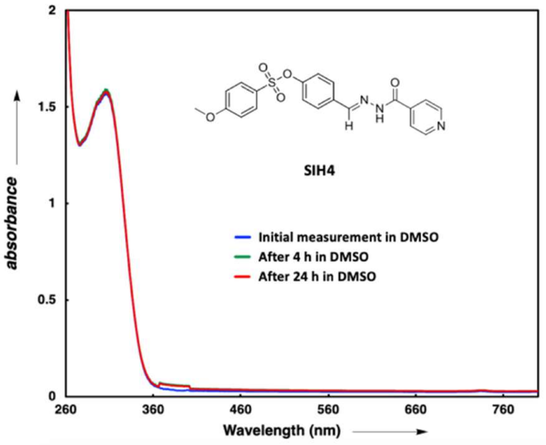

b)

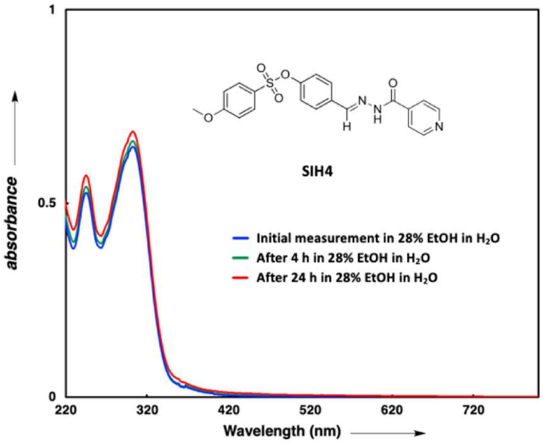

a)

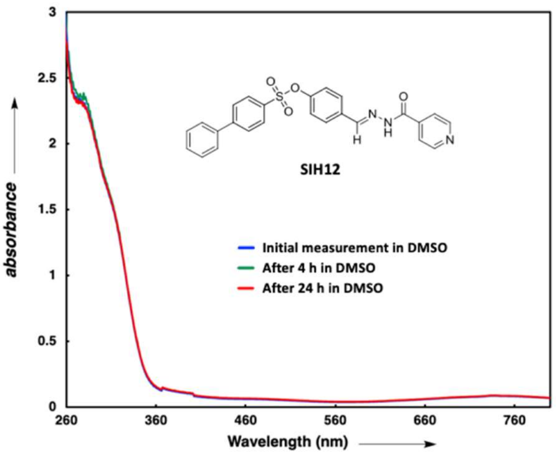

b)

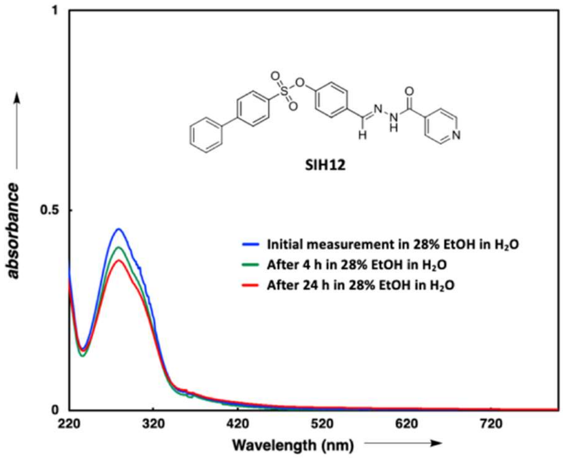

Molecular docking studies

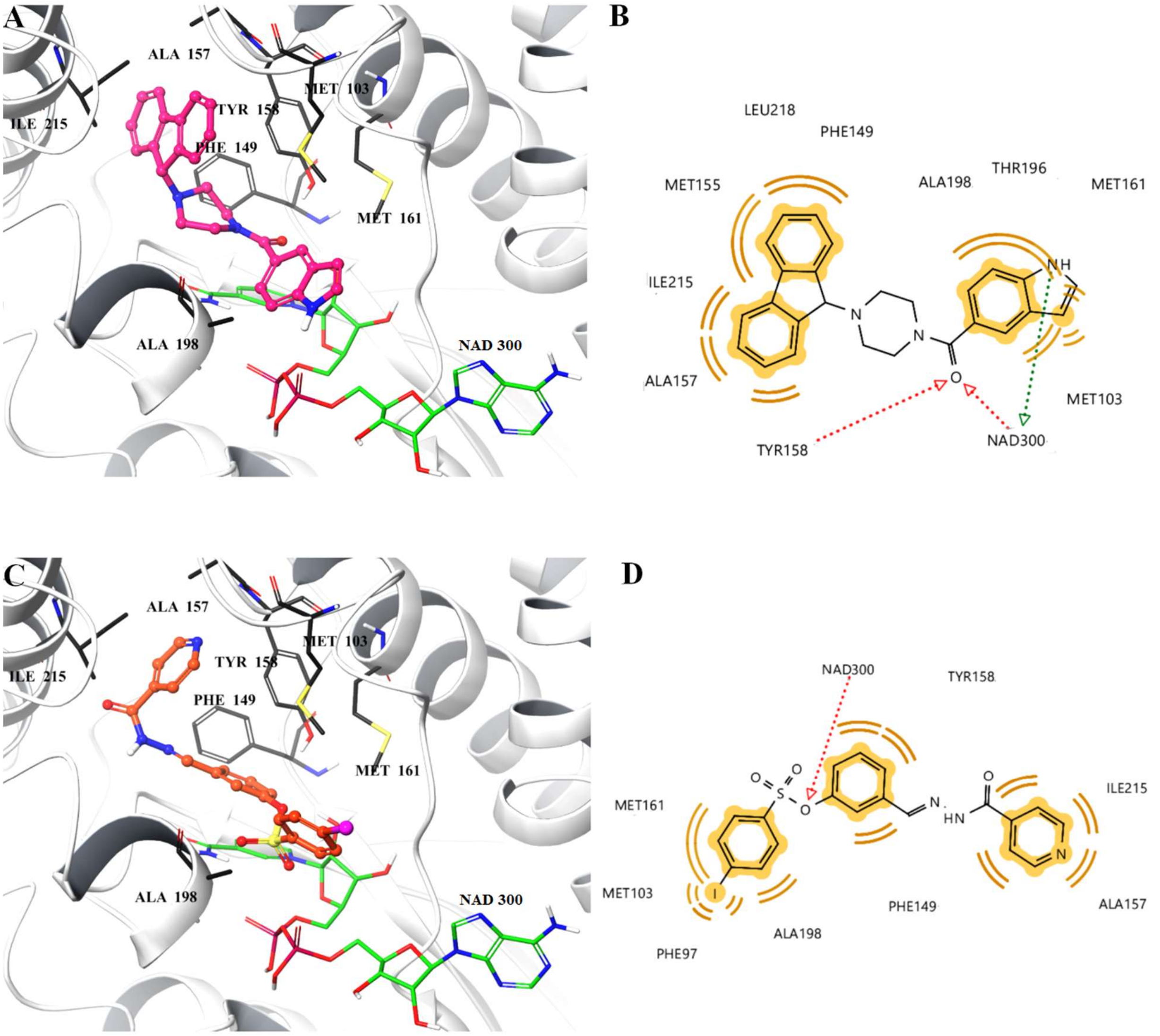

3D view of GEQ (A) and the most plausible binding mode of **SIH7** (C) in the active pocket of InhA (PDB ID:1P44). Ligands are shown as colored balls and sticks, protein backbone as white cartoons, and amino acid residues participating in the binding of both ligands as gray sticks. 2D depictions of enzyme-ligand interactions are provided for GEQ (B) and **SIH7** (D): Hydrogen bond acceptors-red arrows, hydrogen bond donor-green arrow, and hydrophobic interactions-yellow spheres.

Initially, we examined the binding mode of GEQ, the co-crystallized ligand of 1P44. This inhibitor binds to the InhA enzyme through hydrogen bonds with NAD^+ and Tyr158, like most of the direct inhibitors of InhA reported in the literature. Furthermore, two terminal aromatic rings were responsible for forming hydrophobic contacts within the lipophilic pockets of the active site. Subsequently, we analyzed the docking pose of **SIH7** in the same binding pocket. **SIH7** demonstrated a similar orientation with GEQ and formed hydrogen bond with the cofactor NAD through the sulfonate moiety. Additionally, aromatic rings mediated hydrophobic interactions

within the same lipophilic pockets occupied by the co-crystallized ligand. However, hydrogen bond with Tyr158 as one of the key interactions of direct InhA inhibitors lacks for **SIH7**. Consequently, although **SIH7** occupies the native ligand-binding pocket with many common interactions, the lacking hydrogen bond with Tyr158 can explain the moderate InhA inhibitory activity of **SIH7**.
